# Sequence controlled MOF-on-MOF microcrystals for multidomain liquid chromatography stationary phases

**DOI:** 10.1039/d5sc08244g

**Published:** 2025-12-23

**Authors:** Toshiaki Matsumura, Takashi Uemura, Nobuhiko Hosono

**Affiliations:** a Department of Applied Chemistry, Graduate School of Engineering, The University of Tokyo 7-3-1 Hongo, Bunkyo-ku Tokyo 113-8656 Japan uemurat@g.ecc.u-tokyo.ac.jp nhosono@g.ecc.u-tokyo.ac.jp

## Abstract

Designing liquid chromatography (LC) stationary phases with novel separation mechanisms is essential for advancing separation science. Herein, we report hierarchical metal–organic framework (MOF) stationary phases that integrate two distinct MOFs with different pore architectures, enabling multi-domain molecular recognition within a single crystalline material. Specifically [M_2_(bdc)_2_(ted)]_*n*_ (M-BDC; M = Cu or Zn, bdc = 1,4-benzenedicarboxylate, ted = triethylenediamine) and [M_2_(ndc)_2_(ted)]_*n*_ (M-NDC; ndc = 1,4-naphthalenedicarboxylate) are employed as the inner (core) and outer (shell) phases to construct heteroepitaxial MOF-on-MOF architectures within individual crystals. By precisely controlling the growth sequence, MOF-on-MOF layered structures are formed, which are subsequently used as LC stationary phases. The retention behavior and separation performance of these MOF-on-MOF materials are evaluated using polycyclic aromatic hydrocarbons (PAHs) as probe analytes. The stationary phases exhibit distinct retention profiles depending on the heteroepitaxial sequence. In the M-NDC-on-M-BDC system, where the outer shell MOF has smaller pores than the core, the shell functions as an effective recognition layer, strongly influencing the retention behavior of the column even at low shell loading. Conversely, in the M-BDC-on-M-NDC system, where the shell MOF possesses larger pores, analytes can diffuse through the shell and interact with the core MOF, resulting in a reduced impact of the shell on the overall retention. These findings highlight that spatial arrangement and pore hierarchy within MOF-on-MOF architectures critically influence chromatographic behavior. This work demonstrates a new strategy for designing advanced MOF-based LC stationary phases based on modular MOF assembly.

## Abstract 2

Programmable MOF-on-MOF structures offer a modular route to multidomain LC stationary phases, revealing how spatial pore design governs retention and enables new separation selectivity.

## Graphical abstract

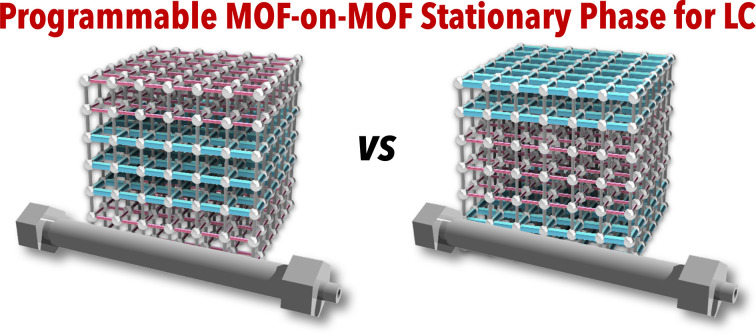

## Introduction

Stationary phases are central to the performance of liquid chromatography (LC), as they determine both the selectivity and efficiency of molecular separations.^1–3^ Over the decades, a variety of separation modes, including adsorption, normal-phase and reversed-phase partitioning, ion exchange, and size exclusion, have been developed to address diverse analytical challenges. Conventional LC stationary phases are most commonly based on spherical silica particles, which are typically surface-modified to tune their interactions with analytes.^4,5^ Among them, octadecylsilyl (ODS)-functionalized silica remains the most widely used material in reversed-phase LC.^6^ Tailored surface functionalization, often through alkyl chains or polar functional groups, has been a foundational strategy for modulating separation selectivity.^7,8^ However, the separation mechanism of each conventional stationary phase is inherently limited to a narrow physicochemical basis such as hydrophobicity or size, restricting their applicability to structurally diverse analytes.

To overcome these limitations, recent efforts have explored mixed-mode stationary phases that integrate multiple retention mechanisms, most commonly combining hydrophobic and ionic interactions, within a single material.^9–11^ These materials have expanded the scope of LC to more complex targets, including biomolecules^12–14^ and synthetic polymers.^15^ Nonetheless, achieving high-resolution separation for analytes with subtle structural or polarity differences, such as positional isomers or polymer mixtures, remains a significant challenge.^16^ To meet these emerging demands, new strategies are required to create stationary phases with spatially and functionally heterogeneous recognition environments.

Metal–organic frameworks (MOFs) have emerged as a promising platform for next-generation stationary phases.^17–19^ Composed of metal ions and organic linkers, MOFs are crystalline porous materials known for their structural diversity and tunable pore environments.^20–22^ Early applications of MOFs in chromatography focused on gas chromatography (GC), where their high surface areas and well-defined adsorption sites were leveraged for efficient separations.^23–26^ These efforts later extended to LC,^27–29^ enabling the separation of hydrocarbons,^30–34^ fullerenes,^35^ aromatic compounds,^36–39^ chiral molecules,^40–44^ and more recently, macromolecules such as synthetic polymers.^45–49^ The designability of MOFs offers precise control over pore size, shape, hydrophobicity, and chemical functionality, making them well-suited for advanced LC separations where analyte recognition requires finely tuned host–guest interactions.^23,46,50^

In addition to single-component MOF stationary phases, multicomponent systems such as mixed-MOF beds and multivariate MOFs (MTV-MOFs, *i.e.* solid-solution, mixed-component MOFs)^51–54^ incorporating multiple linkers have been investigated.^47,55^ These strategies allow for finer modulation of retention behaviors and selectivity profiles. More recently, MOF-on-MOF heterostructures have been developed as hierarchical materials in which one MOF is epitaxially grown on the surface of another.^56–58^ These architectures combine distinct pore environments within a single crystal and have been studied in the context of gas adsorption and separation.^59–61^ In LC applications, MOF-on-MOF structures have been primarily employed to enhance the separation efficiency and physical stability of the core MOF phase.^62^ In these systems, the core MOF typically serves as a complementary porous substrate that improves the interfacial contact for the outer shell MOF, which is mainly responsible for molecular recognition and selectivity.^63,64^ Consequently, these earlier MOF-on-MOF systems have relied on a single recognition domain in terms of selectivity, and the cooperative or synergistic effects of combining two distinct MOF phases have not yet been fully explored. The rational design of stationary phases with spatially separated yet interconnected recognition environments holds significant potential for enabling more versatile analyte discrimination and expanding the functional scope of chromatographic separations.

**Fig. 1:**
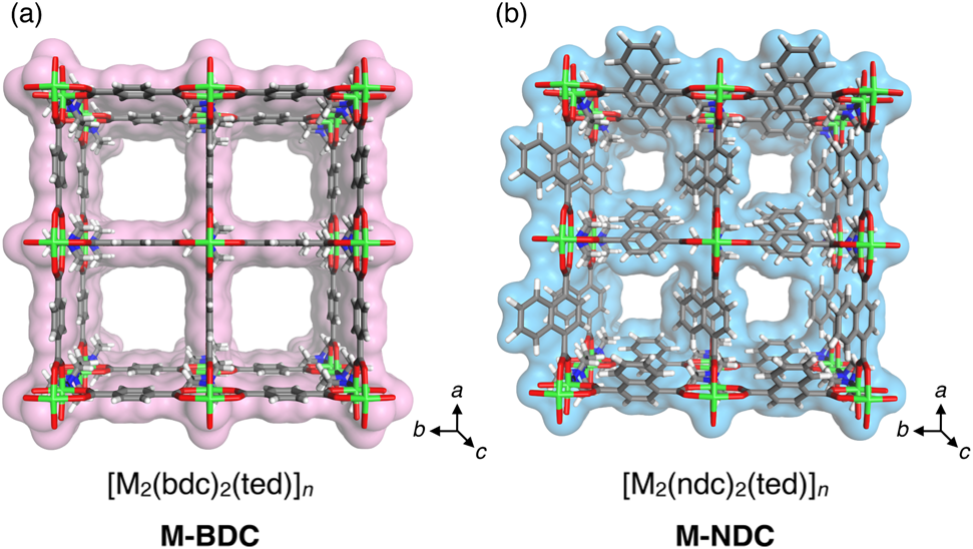
Structures of (a) M-BDC and (b) M-NDC (M = Cu or Zn), composed of bdc and ndc dicarboxylate linkers, respectively. Both MOFs adopt isostructural pillared-layer frameworks with pseudo one-dimensional channels, but exhibit different pore sizes and surface characteristics due to the variation in the dicarboxylate ligand structure. Color code: C, grey; N, blue; O, red; H, white; metal (Cu or Zn), green.

**Fig. 2:**
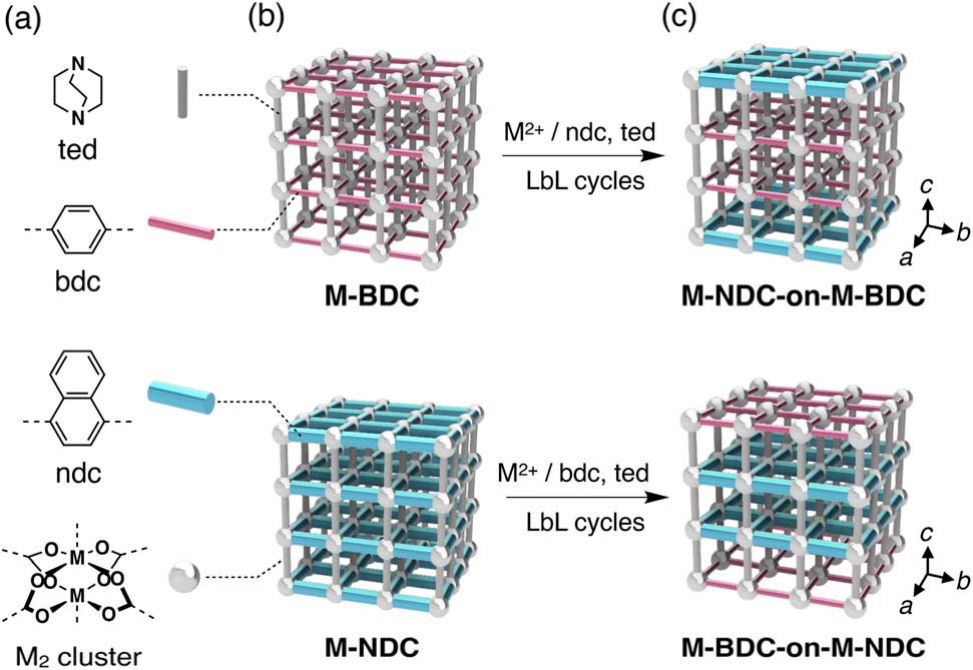
Modular structures of MOF-on-MOF multidomain crystals. (a) Chemical structures of the organic linkers (ted, bdc, and ndc) and the dinuclear paddle-wheel cluster (M_2_ cluster) that interconnects the ligands (M = Cu or Zn). (b) Schematic illustrations of the parent MOFs, M-BDC (top) and M-NDC (bottom). (c) Schematic illustrations of the three-layered MOF-on-MOF architectures obtained by LbL growth of the shell MOF from the core crystal: M-NDC-on-M-BDC (top) and M-BDC-on-M-NDC (bottom) (M = Cu or Zn).

To address this challenge, here we designed MOF-on-MOF stationary phases for LC that sequentially integrate two distinct MOFs [M_2_(bdc)_2_(ted)]_*n*_ (M-BDC, M = Cu of Zn, bdc = 1,4-benzenedicarboxylate, ted = triethylenediamine) and [M_2_(ndc)_2_(ted)]_*n*_ (M-NDC, ndc = 1,4-naphthalenedicarboxylate), into single crystalline particles through heteroepitaxial growth (Fig. 1). By varying the metal ion (M = Zn or Cu) and the dicarboxylate linker (bdc or ndc), we obtained four MOFs with similar topologies but different pore sizes and surface characteristics, all of which exhibit effective analyte retention. Using a layer-by-layer (LbL) approach, we sequentially grew shell MOFs on core MOF crystals to construct three-layered architectures with precisely defined spatial arrangements (Fig. 2). This modular strategy enables the synthesis of hierarchical materials with tunable domain size, composition, and buildup sequence.

The resulting MOF-on-MOF powders were packed into LC columns and evaluated using polycyclic aromatic hydrocarbons (PAHs) as probe analytes. The retention behavior exhibited an intriguing dependence on the core–shell sequence. MOF-on-MOFs with a smaller-pore shell, such as M-NDC-on-M-BDC, exhibited retention behavior primarily governed by the shell MOF. In contrast, materials with a larger-pore shell, such as M-BDC-on-M-NDC, allowed deeper penetration of analytes into the core MOF, resulting in retention profiles more reflective of the core material. These findings demonstrate that MOF-on-MOF architectures serve as a modular platform for engineering stationary phases with controllable spatial recognition domains. This approach introduces a new design concept for LC materials and expands the toolbox for achieving selective and tunable chromatographic separation.

## Results and discussion

### Synthesis of MOF microcrystals and column preparation

In this study, we selected isoreticular pillared-layer-type MOFs, M-BDC (M = Cu or Zn; major pore opening: 7.5 × 7.5 Å^2^)^65,66^ and M-NDC (major pore opening: 5.7 × 5.7 Å^2^),^21,67^ which feature pseudo one-dimensional (1D) channels with systematically tunable pore sizes (Fig. 1). In both the BDC and NDC systems, the major 1D pore channels are oriented along the *c*-axis of the crystal, perpendicular to the layers formed by the dicarboxylate ligands. It should be noted that the effective pore opening of the NDC-based frameworks is estimated to fluctuate around 5.7 Å, taking into account the dynamic rotational motion of the ndc ligands.

These pillared-layer-type MOFs allow fine modulation of pore dimensions by variation of the dicarboxylate linker, making them suitable candidates for constructing MOF-on-MOF architectures with distinct but structurally compatible pore environments.^68,69^ Both M-BDC and M-NDC have been previously reported for the adsorption of PAHs^70,71^ and demonstrated promise as LC stationary phases.^45–49,72^

To establish a reference for retention behavior, we first synthesized single-phase, parent MOFs (Zn-BDC, Zn-NDC, Cu-BDC, and Cu-NDC) *via* solvothermal reactions in DMF (see Experimental section and SI). The resulting microcrystalline particles had average sizes ranging from 3 to 11 µm (Table S1). The MOF particles were slurry-packed into stainless-steel columns (I. D. = 4 mm, L. = 50 mm), following standard LC column preparation protocols. Details of the synthetic procedures and column packing methods are provided in Experimental section.

LC retention behavior was investigated using perylene as a model PAH analyte (Fig. S1). Before the LC experiments, we evaluated the batch adsorption capability of the parent MOFs, which confirmed appreciable uptake of perylene in both Zn-BDC and Zn-NDC (Fig. S2 and SI). The molecular dimensions of perylene (8.94 Å × 11.3 Å, Fig. S1) apparently approach the upper limit of Zn-NDC channel size, even considering its dynamic ligand rotation. This narrow pore size has a pronounced effect on diffusion kinetics. Compared to Zn-BDC, which possesses larger channels, Zn-NDC exhibited slower adsorption rates (Fig. S2). The reduced diffusion rate in Zn-NDC is attributed to its smaller pore aperture, which imposes greater steric constraints on analyte transport compared to the wider pores of Zn-BDC.

**Fig. 3:**
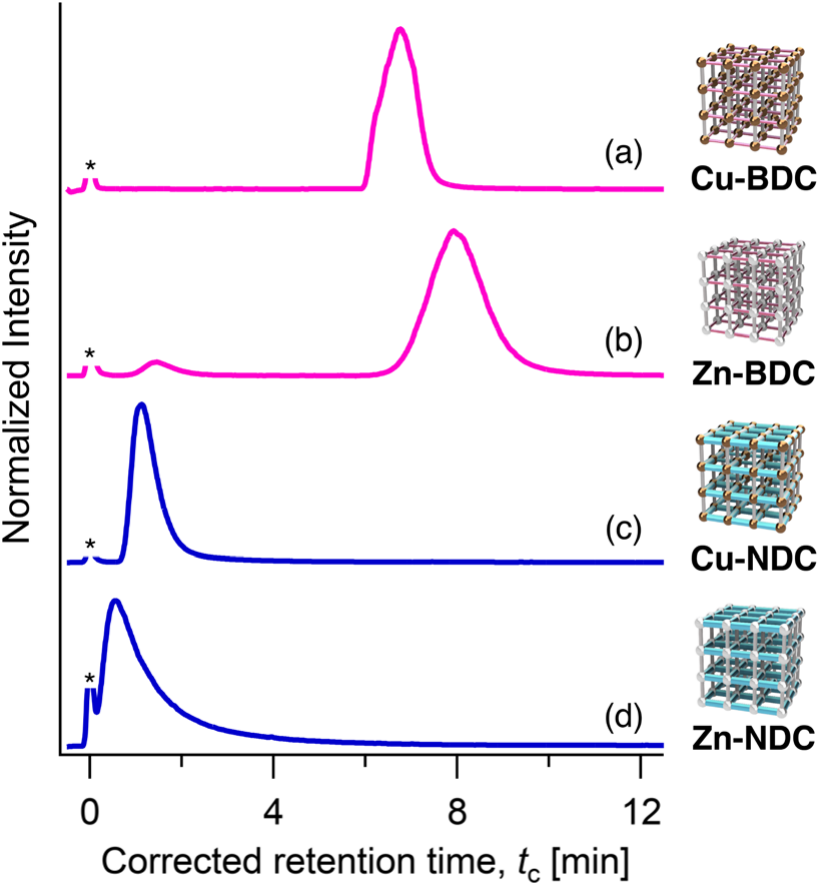
HPLC chromatograms of perylene on (a) Cu-BDC, (b) Zn-BDC, (c) Cu-NDC, and (d) Zn-NDC packed columns, recorded using an evaporative laser scattering detector (ELSD). An asterisk denotes the front injection peak, which was used to determine the *t*_0_ point. Eluent: hexane, temperature: 40 °C, flow rate: 1.0 mL min^−1^.

HPLC measurements were performed in hexane at 40 °C with a flow rate of 1.0 mL min^−1^ (see Experimental section). The retention profiles of the individual single-phase MOF columns revealed that the interaction strength between perylene and the column was governed primarily by pore size and linker structure (bdc *vs.* ndc), through potential π–π interactions,^70^ rather than by the metal center (Cu or Zn) (Fig. 3).

Among the MOFs tested, M-BDC exhibited higher retention for perylene compared to M-NDC (Fig. 3). This trend is attributed to the pore size effect discussed earlier, where the larger channel of M-BDC permits more facile diffusion and deeper penetration of the perylene molecule, resulting in enhanced retention. In contrast, the smaller pore aperture of M-NDC restricts diffusion, leading to shorter retention. Although the molecular dimensions of perylene slightly exceed the estimated pore opening of M-NDC, we consider that the rotational flexibility of the ndc ligands allows transient widening of the pore openings, thereby enabling the adsorption and desorption of perylene within the pores. This retention trend is consistent with our previous studies, where kinetic contributions arising from pore accessibility significantly influenced the retention strength and separation performance of MOF-based stationary phases.^47,49^ These findings motivated us to explore whether combining M-BDC and M-NDC within a single MOF-on-MOF particle could enable systematic modulation of retention behavior through spatial control of pore sequence.

### Synthesis of MOF-on-MOF microcrystals through the layer-by-layer method

We first attempted the synthesis of Cu-NDC-on-Zn-BDC, in which a shell of Cu-NDC is grown on preformed Zn-BDC core crystals. Our initial strategy involved subjecting Zn-BDC microcrystals to a conventional solvothermal reaction using Cu-NDC precursors (see SI). Powder X-ray diffraction (PXRD) of the product matched that of the parent core MOFs, consistent with their isoreticular crystal structures and indicating the absence of any new or impurity phases (Fig. S3). However, scanning electron microscopy with energy-dispersive X-ray spectroscopy (SEM-EDX) elemental mapping revealed broad particle size and shape distributions along with homogeneous Cu and Zn distributions throughout the individual crystals, suggesting that no core–shell structure was formed (Fig. S4).

**Fig. 4:**
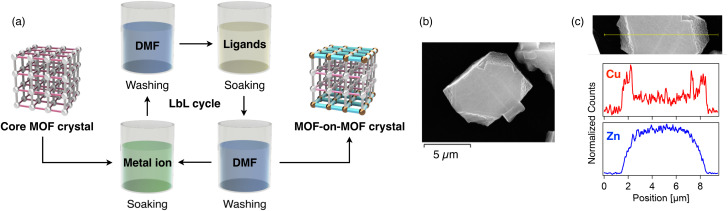
(a) Schematic illustration of the synthesis of MOF-on-MOF crystals through the LbL method. (b) A SEM image of a representative crystal of Cu-NDC-on-Zn-BDC (*x*_NMR_ = 0.10). (c) EDX line-scan profile (bottom) showing the distribution of Cu (red) and Zn (blue) along the yellow line indicated in the corresponding SEM image (top).

To enable controlled heteroepitaxial growth, we adopted the LbL approach,^73^ which permits gradual shell growth over the core MOF through iterative cycles of metal-ion and ligand exposures (Fig. 4a). Zn-BDC core crystals were alternately immersed in a DMF solution of Cu^2+^ ions and a DMF solution containing ndc and ted ligands, with thorough DMF washing between each step. Repeating this metal–ligand cycle led to the gradual deposition of Cu-NDC on the surface of Zn-BDC crystals (see Experimental section and SI). Using Cu^2+^, ndc, and ted each at a concentration of 1.25 mM, five LbL cycles were performed. The resulting crystals were vacuum-dried at 120 °C overnight to yield Cu-NDC-on-Zn-BDC microcrystals.

PXRD patterns of the Cu-NDC-on-Zn-BDC samples were identical to the individual pure MOFs, confirming the phase purity after shell growth (Fig. S5). SEM imaging showed that the original rectangular morphology of Zn-BDC was largely retained, although the Cu-NDC-on-Zn-BDC microcrystals appeared slightly truncated (Fig. 4b). EDX line-scan analysis across individual particles revealed a core–shell architecture, with Zn concentrated at the center and Cu localized near the particle edges (Fig. 4c). The thickness of shell MOF was observed to be several hundred nm. Multiple EDX line scans indicated that Cu preferentially accumulated along the short side edges, suggesting preferential shell growth on the 001 facets (Fig. S6). This anisotropic growth of Cu-NDC along the *c*-axis is consistent with previous reports^74,75^ and enables the formation of three-layered MOF-on-MOF architectures (Fig. 2c). The observed truncation of the crystal edges may be due to partial etching of the Zn-BDC core during the LbL process, a phenomenon frequently associated with surface-energy minimization (Fig. S6).^76^ A visible boundary at the particle surface also supported the presence of a Cu–Zn interface (Fig. S6).

The shell MOF fraction among the entire MOF-on-MOF crystals was quantified by both ^1^H NMR and X-ray fluorescence (XRF) spectroscopy. ^1^H NMR analysis, performed after acid digestion in a DMSO-*d*_6_/DCl mixture, yielded a shell-ligand fraction of *x*_NMR_ = 0.10 (Fig. S7, Table S2). XRF analysis gave a shell-metal fraction of *x*_XRF_ = 0.12 (Table S2), indicating good agreement between ligand and metal ratios and confirming successful shell growth. Hereafter, the NMR-based value, *x*_NMR_, is adopted to represent the shell MOF fraction since PAH separation is predominantly governed by ligand chemistry. The integral ratio of carboxylate to ted in ^1^H NMR spectra was always 2 : 1, suggesting a negligible presence of unreacted ligands or amorphous content in the MOFs.

For comparison, we also synthesized a solid-solution (mixed-ligand) MOF by co-assembling ndc and bdc ligands using the same metal and ligand fractions as in the Cu-NDC-on-Zn-BDC (shell fraction: *x*_NMR_ = 0.10) sample (Fig. S8 and SI). The resulting solid-solution material exhibited color characteristics and micromorphologies that were entirely different from those of the MOF-on-MOF crystals (Fig. S9 and S10). These distinctions support the formation of heteroepitaxial MOF-on-MOF structures rather than mixed-ligand solid solutions.

Particle size distribution analysis on the Cu-NDC-on-Zn-BDC samples showed an increase in particle size after LbL treatment, consistent with shell layer formation (Table S1). N_2_ adsorption measurements at 77 K were conducted to evaluate the porosity of pristine single-phase MOFs and the MOF-on-MOF sample. The individual Cu-NDC and Zn-BDC exhibited Brunauer–Emmett–Teller (BET) surface areas of 1129 and 1989 m^2^ g^−1^, respectively, with maximum uptakes of 259 and 457 cm^3^ g^−1^ (STP) at *p*/*p*_0_ = 0.99 (Fig. S11), which are consistent with the literature values.^21,77^Cu-NDC-on-Zn-BDC (*x*_NMR_ = 0.10) showed intermediate properties with a BET surface area of 1698 m^2^ g^−1^ and uptake of 390 cm^3^ g^−1^ (STP), supporting the formation of a hybrid pore system.

To further understand the growth process, the number of LbL cycles was varied (1, 3, and 5 cycles) using 1.25 mM precursor concentrations (see Experimental section and SI). PXRD patterns confirmed structural integrity for all products (Fig. S12). SEM-EDX analysis showed negligible Cu content after 1 cycle, indicating minimal shell growth (Fig. S13). In contrast, after 3 and 5 cycles, Cu was clearly localized at the surface and Zn remained at the core, indicating successful formation of heteroepitaxial core–shell architectures. ^1^H NMR analysis for acid-digested samples showed increasing *x*_NMR_ values from 0.032 (1 cycle) to 0.10 (3 cycles), with saturation at 0.10 after 5 cycles (Fig. S14), suggesting that 5 cycles are sufficient under these conditions.

Having optimized the LbL cycle number and conditions, we next synthesized Cu-NDC-on-Zn-BDC samples using different precursor concentrations (1.25, 2.5, and 12.5 mM; 5 cycles each). PXRD (Fig. S15) and SEM-EDX (Fig. S16) analyses confirmed the successful synthesis of MOF-on-MOF architectures. ^1^H NMR gave *x*_NMR_ = 0.10, 0.15, and 0.26 for the samples synthesized under 1.25, 2.5, and 12.5 mM precursor concentrations, respectively, indicating that higher precursor concentrations increased the shell fraction (Fig. S17 and Table S2). XRF gave *x*_XRF_ = 0.12, 0.15, and 0.38, respectively. While the low-concentration samples showed agreement between ligand and metal ratios, higher precursor concentrations resulted in higher *x*_XRF_ values than *x*_NMR_, suggesting partial Cu^2+^/Zn^2+^ ion exchange during the LbL process. This is reasonable, as Cu^2+^ is known to exchange with Zn^2+^ in Zn-BDC during immersion in Cu^2+^-containing solutions.^78,79^ Particle size remained in the range of 8 to 11 µm for all samples, with appreciable increases after shell formation (Table S1). N_2_ adsorption studies showed decreasing BET surface area and maximum uptake with increasing shell content, consistent with the smaller pore size of Cu-NDC relative to Zn-BDC (Fig. S11).

Using identical LbL protocols, we also synthesized all-Zn MOF-on-MOF crystals, Zn-NDC-on-Zn-BDC, employing precursor concentrations of 2.5 mM and 12.5 mM (see SI). PXRD confirmed the integrity of crystalline frameworks (Fig. S18). ^1^H NMR analysis revealed *x*_NMR_ = 0.11 and 0.29 for the two samples, respectively (Fig. S19). Owing to the absence of Cu^2+^, confocal laser scanning microscopy (CLSM) successfully visualized the core–shell structure, in which the non-fluorescent Zn-BDC core was clearly distinguished from the bright Zn-NDC-fluorescent shell (Fig. S20). These results confirm the spatial segregation of bdc and ndc ligands within the MOF-on-MOF crystals. It should be noted that such ligand-domain segregation could not be visualized in the Cu–Zn systems due to fluorescence quenching by Cu^2+^,^80,81^ which limited the utility of CLSM in those cases.

Subsequently, we synthesized the inverse pore sequence, Cu-BDC-on-Zn-NDC, by reversing the ligands in the core and shell phases under otherwise identical LbL conditions (1.25, 2.5, and 12.5 mM; 5 cycles each) (see SI). The porous properties of the materials were characterized by N_2_ adsorption analyses (Fig. S11). SEM images revealed that the rectangular morphology of the Zn-NDC core was largely retained, although some particles showed deformation and a reduced particle size than that expected by the LbL shell growth reaction (Fig. S21 and Table S1). The MOF-on-MOF architectures were characterized by PXRD (Fig. S22) and SEM-EDX (Fig. S23) analyses. ^1^H NMR analysis yielded *x*_NMR_ = 0.10, 0.21, and 0.53, while XRF gave *x*_XRF_ = 0.11, 0.23, and 0.36 for the same set of samples (Fig. S24 and Table S2). At higher precursor concentrations, the ligand content exceeded the metal content, likely due to ligand exchange during the LbL process.^82,83^ In this case, the smaller bdc ligands likely replaced bulkier ndc ligands in the Zn-NDC core, a process facilitated by the high ligand concentration.^84^ The morphology loss observed under SEM and the reduced particle size after the LbL process further support this interpretation.

The monometallic analogues of the Cu-BDC-on-Zn-NDC system, namely Zn-BDC-on-Zn-NDC, were also synthesized using precursor concentrations of 2.5 mM and 12.5 mM, which yielded samples with *x*_NMR_ = 0.16 and 0.71, respectively (Fig. S25 and S26). CLSM analysis confirmed the expected inverted sequence, showing the fluorescent Zn-NDC core enclosed on both sides by non-fluorescent Zn-BDC layers (Fig. S27).

### Retention behavior of MOF-on-MOF stationary phases

**Fig. 5:**
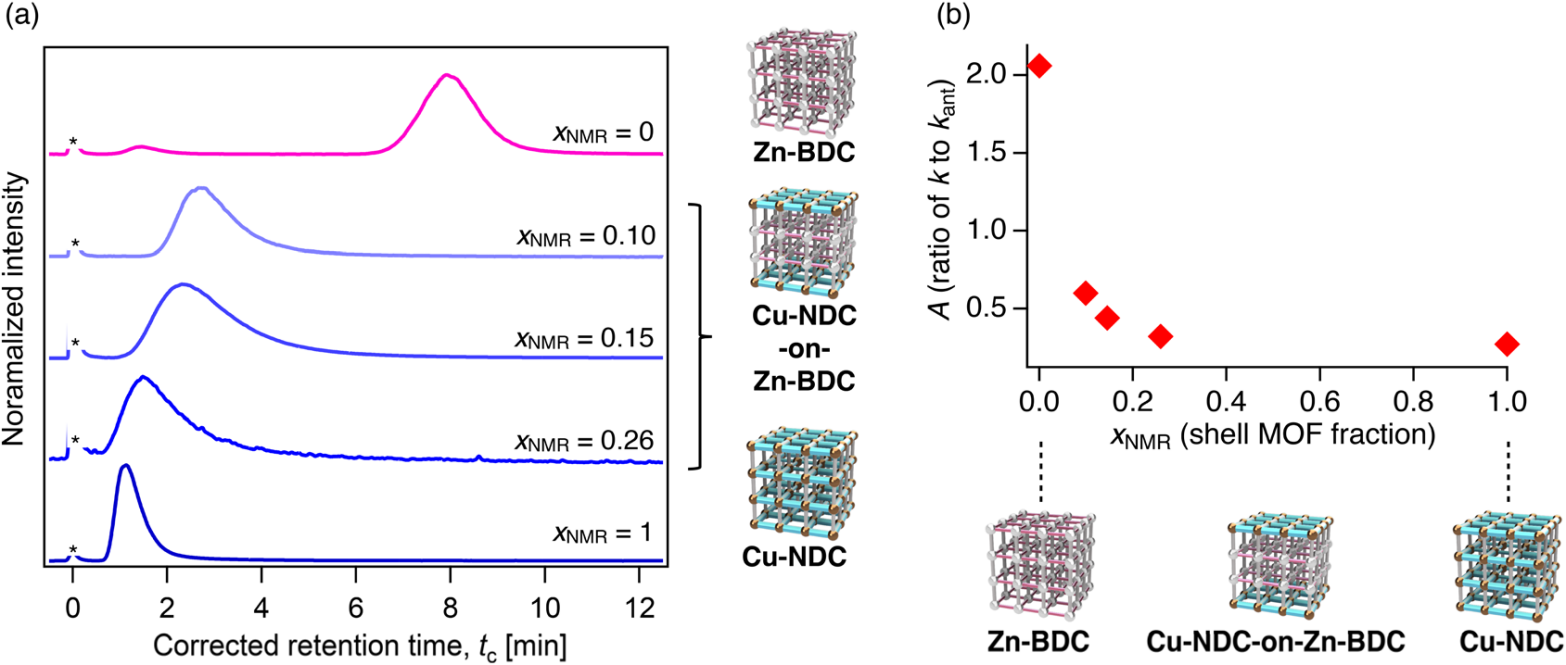
(a) HPLC chromatograms of perylene on Cu-NDC-on-Zn-BDC columns with varying shell fractions (*x*_NMR_ = 0.10, 0.15, and 0.26). Columns with *x*_NMR_ = 0 and 1 correspond to the parent MOFs Zn-BDC and Cu-NDC, respectively. An asterisk denotes the front injection peak, which was used to determine the *t*_0_ point. (b) Retention factor ratio *A* (defined as *k*/*k*_ant_, where *k*_ant_ is the retention factor of anthracene) plotted as a function of shell fraction *x*_NMR_. Eluent: hexane, temperature: 40 °C, flow rate: 1.0 mL min^−1^, detector: ELSD.

We evaluated the chromatographic performance of MOF-on-MOF stationary phases by packing them into the LC columns and conducting retention measurements at 40 °C using hexane as the mobile phase. We first examined the M-NDC-on-M-BDC systems. We prepared Cu-NDC-on-Zn-BDC series, in which the shell fractions were varied as 0.10, 0.15, and 0.26. Perylene was selected as a probe analyte. Distinct retention peaks were observed for all Cu-NDC-on-Zn-BDC columns (Fig. 5a). In each case, the retention time (*t*_R_, corrected retention time; see Experimental section) appeared between those of the single-phase pure MOF (Cu-NDC and Zn-BDC) columns. Increasing the thickness of the Cu-NDC shell progressively decreased *t*_R_, shifting retention behavior closer to that of the shell MOF (Fig. 5a). These results suggest that both the core and shell MOFs contribute to analyte retention. Notably, even a small fraction of shell MOF in the Cu-NDC-on-Zn-BDC (shell fraction: *x*_NMR_ = 0.10) induced significant changes in retention (Fig. 5a), indicating that the outer recognition domain exerts a dominant influence.

To eliminate potential effects arising from column-to-column differences in packing density or particle size, retention behavior was normalized using a retention factor ratio *A*, defined as *A* = *k*/*k*_ant_, where *k* is the retention factor of the analyte and *k*_ant_ is that of anthracene, used as a standard (Fig. 5b and S28, Tables S3 and S4). The *A* values for perylene and phenanthrene were measured for each MOF-on-MOF column. For perylene, *A* decreased with increasing shell MOF fraction, consistent with greater contributions from the Cu-NDC shell (Fig. 5b). For phenanthrene, the pure Zn-BDC column showed modest retention with observable elution, while Cu-NDC led to strong adsorption without elution (Fig. S29). Similarly, the heteroepitaxial Cu-NDC-on-Zn-BDC stationary phases also exhibited strong retention for phenanthrene, with no elution peaks observed, suggesting that the retention was predominantly governed by the Cu-NDC shell, even at low shell content (Fig. S29). These results confirm that analyte interactions are highly sensitive to the outer pore environment.

**Fig. 6:**
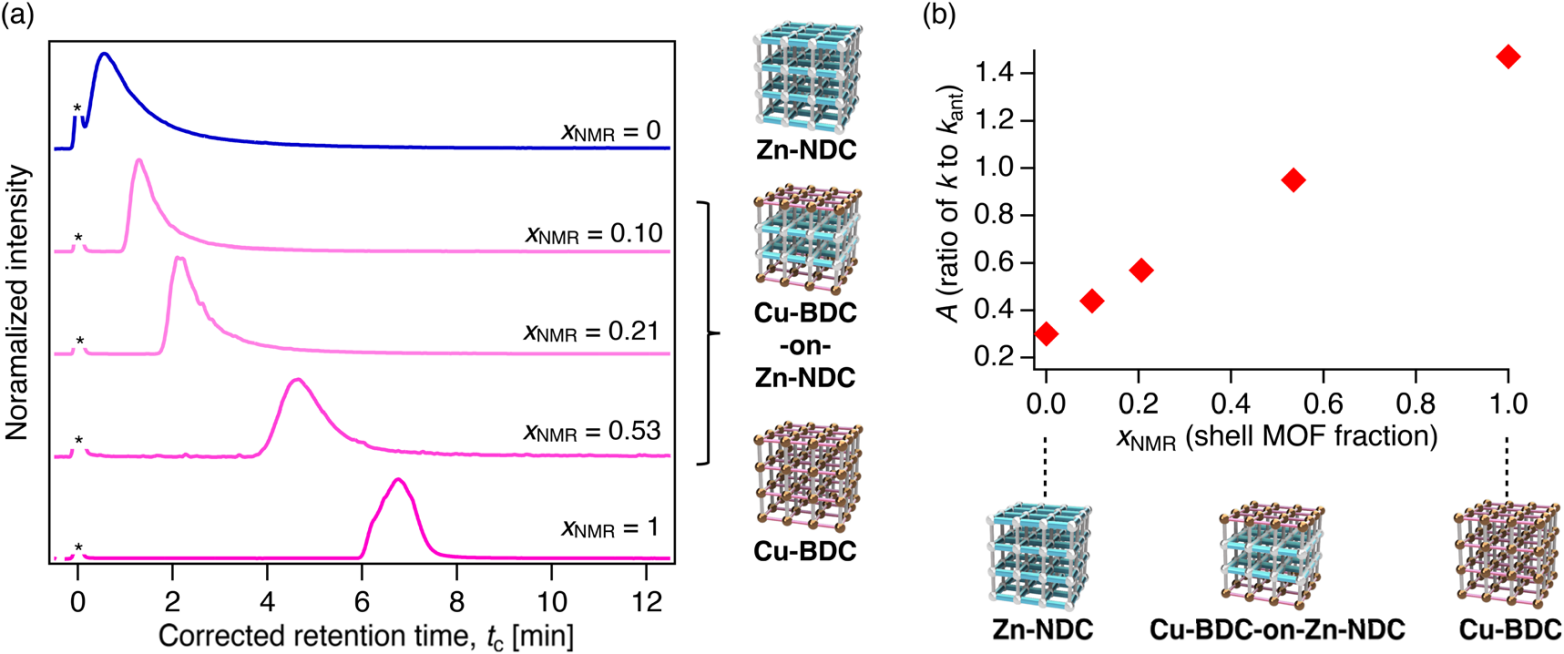
(a) HPLC chromatograms of perylene on Cu-BDC-on-Zn-NDC columns with varying shell fractions (*x*_NMR_ = 0.10, 0.21, and 0.53). Columns with *x*_NMR_ = 0 and 1 correspond to the parent MOFs Zn-NDC and Cu-BDC, respectively. An asterisk denotes the front injection peak, which was used to determine the *t*_0_ point. (b) Retention factor ratio *A* (defined as *k*/*k*_ant_, where *k*_ant_ is the retention factor of anthracene) plotted as a function of shell fraction *x*_NMR_. Eluent: hexane, temperature: 40 °C, flow rate: 1.0 mL min^−1^, detector: ELSD.

Finally, we investigated the inverse core–shell configuration, namely M-BDC-on-M-NDC systems. Three packed columns were prepared using the Cu-BDC-on-Zn-NDC samples with shell fractions of 0.10, 0.21, and 0.53, and their LC retention behaviors were evaluated using perylene and phenanthrene as the analytes. For perylene, retention peaks again appeared between those of the parent pure MOF columns (Fig. 6a). Due to the inverted MOF layer sequence, increasing shell thickness led to longer *t*_R_, with retention shifting toward that of the pure Cu-BDC column. Interestingly, the core MOF was influential on retention, as also reflected in the *A* values, which exhibited a monotonic, proportional trend with increasing shell fraction (Fig. 6b, Tables S5 and S6). This behavior contrasts sharply with the exponential-like dependence observed in the Cu-NDC-on-Zn-BDC system (Fig. 5b). A similar monotonic trend was observed for phenanthrene (Fig. S30). These results indicate that, in the Cu-BDC-on-Zn-NDC system, a relatively thick shell is necessary for the retention behavior to approach that of the pure Cu-BDC phase.

It should be noted that the monometallic series of the MOF-on-MOF columns showed PAH retention trends consistent with those observed for the bimetallic systems (Fig. S31 and S32, Tables S7–S10). This agreement indicates that linker structure, rather than metal identity, plays the dominant role in governing retention in the present MOF-on-MOF systems.

**Fig. 7:**
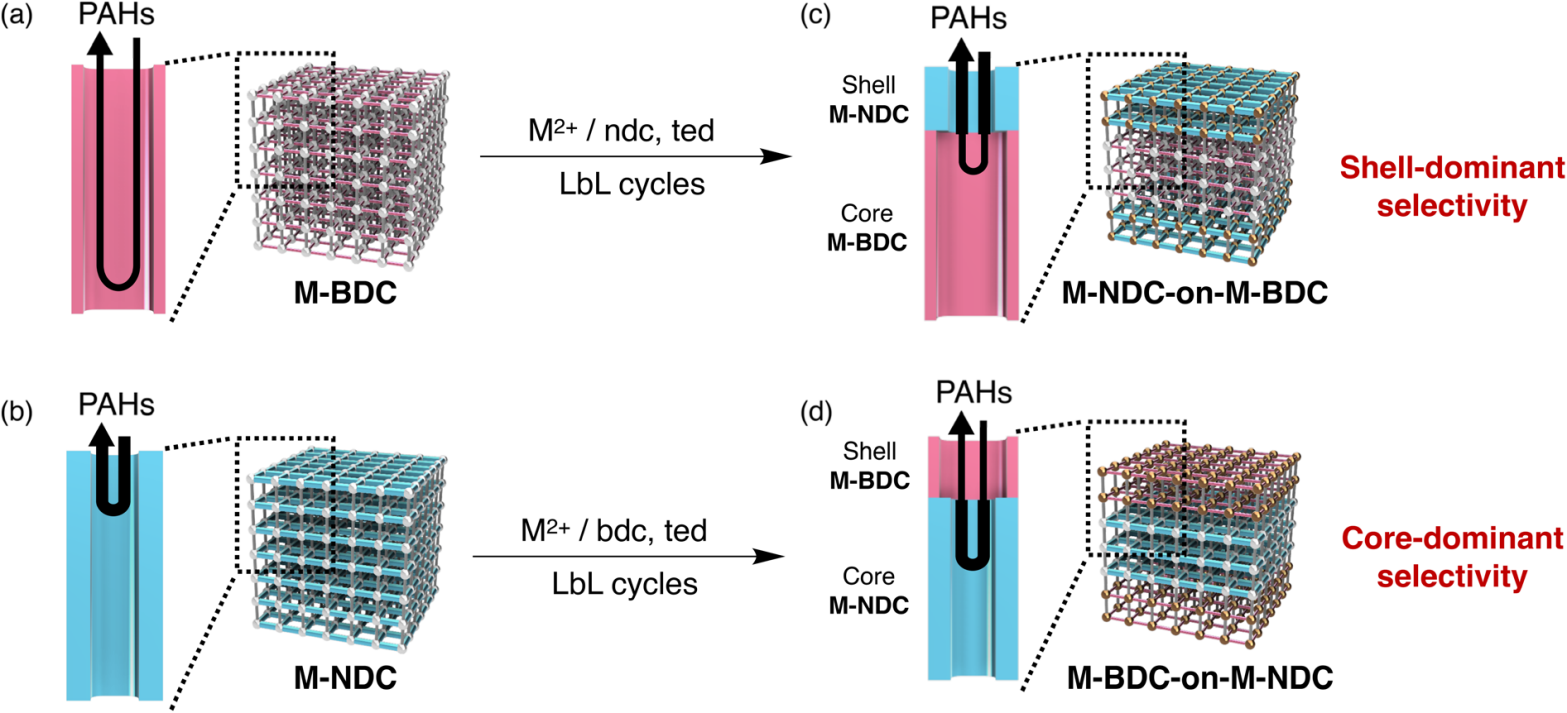
Schematic illustration of adsorption behavior at the surface of (a) M-BDC, (b) M-NDC, (c) M-NDC-on-M-BDC, and (d) M-BDC-on-M-NDC stationary phases. The thickness of the black arrows represents the relative strength of adsorption interactions between the analyte and the MOF channels. (a) PAHs interact moderately with the large pores of M-BDC and can diffuse deeply into the framework. (b) In contrast, PAHs interact strongly with the smaller pores of M-NDC but penetrate only shallowly due to restricted diffusion. (c) In the M-NDC-on-M-BDC architecture, even a thin shell of M-NDC effectively retains PAHs due to strong interactions. (d) In the inverse configuration, M-BDC-on-M-NDC, the large-pore M-BDC shell interacts weakly with PAHs, allowing them to access and be retained by the inner M-NDC core.

Based on the above systematic investigations, we attribute the distinct difference in retention behavior between the M-NDC-on-M-BDC and M-BDC-on-M-NDC systems to the relative pore sizes of the constituent MOFs (Fig. 7). Fig. 7 illustrates the adsorption and desorption behavior of analytes within the MOF channels. In M-BDC, the larger pore size facilitates rapid diffusion, allowing analytes to penetrate more deeply into the framework.^85^ However, the wider pore space results in weaker confined-space interactions; in other words, analyte–pore interactions per unit thickness become relatively moderate.^86^ In contrast, M-NDC features smaller pores that restrict diffusion, leading to shallower infiltration. At the same time, the tighter confinement enhances analyte–framework interactions per unit thickness. As a result, even a thin layer of M-NDC can exert a strong influence on analyte retention.

This interplay between diffusion kinetics and spatial confinement explains the observed trends in MOF-on-MOF stationary phases. In the M-NDC-on-M-BDC configuration, the outer M-NDC shell provides strong analyte interactions, dominating retention behavior even at low shell thickness. Conversely, in the M-BDC-on-M-NDC system, the large-pore M-BDC shell permits analytes to pass through and interact with the small-pore core, resulting in core-dominated retention unless the shell becomes sufficiently thick.

These results collectively demonstrate that MOF-on-MOF stationary phases can exhibit either shell-dominant or core-dominant retention behavior, depending on the layering sequence as well as relative pore sizes and thicknesses of the constituent MOF domains. In particular, small-pore shells act as effective outer recognition layers, strongly affecting analyte interaction even at low loading. In contrast, large-pore shells permit analyte penetration into the core, reducing their impact on overall retention. This ability to program spatial recognition hierarchies represents a significant advantage of the MOF-on-MOF architecture in chromatographic separation.

We note that the present MOF columns exhibit the potential to separate the tested PAH mixtures (Fig. S33); however, their resolution remains limited, likely due to irregular bed morphologies in the column. Achieving higher resolution will require further refinement of MOF particle shape and a narrower particle size distribution.^87^ In addition, this study focused on representative PAHs for which size discrimination and π–π interaction are the primary retention contributors in the current MOF systems. Expanding the analyte library will be an important next step toward evaluating its applicability in more practical separation scenarios.

## Conclusions

In summary, we have successfully fabricated MOF-on-MOF microcrystals with distinct and hierarchical pore environments using an LbL growth strategy. By tuning the precursor concentrations during synthesis, we obtained a series of core–shell MOF structures with systematically varied shell-to-core ratios and demonstrated their application as multidomain stationary phases for LC. Chromatographic evaluation revealed that both the core and shell MOFs contribute to analyte retention, with the relative influence governed by the pore size and spatial arrangement of each domain. When a small-pore shell MOF was grown on a large-pore core MOF, the shell provided dominant molecular recognition, significantly affecting retention even at low shell thickness. In contrast, when the shell MOF had a larger pore size than the core, analytes could access the core more readily, resulting in retention behavior dominated by the inner MOF unless the shell was sufficiently thick. This systematic control over spatial recognition behavior underscores the utility of hierarchical MOF architectures for chromatographic separations. The modular nature of the MOF-on-MOF design provides a versatile platform for tailoring retention properties, offering new opportunities to construct next-generation LC stationary phases with programmable selectivity and multifunctional separation capabilities.

## Experimental section

### Synthesis of MOF-on-MOF microcrystals

Bulk microcrystals of the core MOFs, Zn-BDC and Zn-NDC, were synthesized following previously reported procedures (see SI).^21,45,88^ MOF-on-MOF architectures were fabricated using a layer-by-layer (LbL) growth method adapted from the literature.^73,89^ As a representative procedure, Zn-BDC (500 mg) was dispersed in a 1.25 mM DMF solution of copper(ii) nitrate hydrate and stirred at 120 °C for 15 min. After the reaction, the mixture was centrifuged to remove the supernatant, and the resulting solid was washed three times with fresh DMF using decantation–centrifugation cycles. The solid was then suspended in a 1.25 mM DMF solution containing H_2_ndc and ted, stirred at 120 °C for 30 min, and centrifuged. After three additional DMF washing steps, this two-step cycle was repeated for the desired number of iterations. The final product was vacuum-dried at 120 °C overnight to afford Cu-NDC-on-Zn-BDC. The shell ligand fraction, *x*_NMR_, was determined to be 0.10 based on ^1^H NMR analysis of the sample digested in a DMSO-*d*_6_/DCl (9 : 1, v/v) mixture. Additional synthesis procedures and characterization methods are described in the SI.

### Preparation of MOF-packed columns and LC measurements

MOF-packed columns were prepared according to previously reported methods.^49^ A stainless-steel column (4.0 mm I.D. × 50 mm L., GL Sciences) was packed with approximately 250 mg of MOF-on-MOF powder using hexane as the packing solvent. A slurry of the MOF particles in hexane was introduced into the column using a Shimadzu LC-20AD pump under a packing pressure of approximately 10 MPa. PXRD analysis confirmed that the structural integrity of the MOF was maintained under these conditions. The packed columns were connected to a Shimadzu Prominence HPLC system equipped with an evaporative light scattering detector (ELSD-LTII, Shimadzu). Hexane was used as the mobile phase at a flow rate of 1.0 mL min^−1^. PAH analytes were dissolved in hexane at a concentration of 1 mg mL^−1^, and a 10–50 µL aliquot was injected into the column. The ELSD signal was recorded at a sampling rate of 2 Hz. The corrected retention time, *t*_c_, is defined as *t*_c_ = *t*_R_ − *t*_0_, where *t*_R_ is the observed retention time of the analyte and *t*_0_ is the column hold-up time. The value of *t*_0_ was determined from the front injection peak observed in each chromatogram. The retention factor, *k*, was determined as *k* = (*t*_R_ − *t*_0_)/*t*_0_.

## Author contributions

Conceptualization: N. H., T. U., methodology: T. M., N. H. investigation: T. M., N. H., data acquisition: T. M., data curation: T. M., N. H., supervision: N. H., T. U., writing—original draft: T. M., N. H., writing—review & editing: N. H., T. U.

## Conflicts of interest

There are no conflicts to declare.

## Supplementary Material

SC-OLF-D5SC08244G-s001

## Data Availability

All the data supporting the findings of this study are available in the article and the supplementary information (SI). Supplementary information: synthesis of MOF microcrystals, LbL procedures, batch adsorption experiments, particle size distributions, retention factors, chromatograms, and characterization data, including PXRD, ^1^H NMR, SEM-EDX, CLSM, and gas adsorption profiles. See DOI: https://doi.org/10.1039/d5sc08244g.
