# Supplementary material for: Sequence controlled MOF-on-MOF microcrystals for multidomain liquid chromatography stationary phases
Source: Chem Sci. 2025 Dec 23;17(8):4097–106. doi: 10.1039/d5sc08244g (PMC12766741; doi:10.1039/d5sc08244g)
Supplement: SC-017-D5SC08244G-s001 [file SC-017-D5SC08244G-s001.pdf]

Supplementary Information (SI) for Chemical Science.
This journal is © The Royal Society of Chemistry 2026

Supplementary Information for

Sequence Controlled MOF-on-MOF Microcrystals for Multidomain Liquid Chromatography Stationary Phases

Toshiaki Matsumura,[a] Takashi Uemura,*[a] Nobuhiko Hosono*[a]

[a] Department of Applied Chemistry, Graduate School of Engineering, The University of Tokyo, 7-3-1 Hongo, Bunkyo-ku, Tokyo 113-8656, Japan

1. General Instruments

^{1}H nuclear magnetic resonance (NMR) spectra were recorded using a Bruker Avance III HD spectrometer equipped with a PABBO probe operating at 500 MHz. Powder X-ray diffraction (PXRD) data were recorded on a Rigaku model SmartLab X-ray diffractometer using CuKα radiation in Bragg-Brentano (BB) reflection mode unless otherwise stated. Scanning electron microscopy (SEM) measurements were performed using a Hitachi model SU-5000 at an accelerating voltage of 15 kV. Samples were deposited on a conducting carbon tape attached on a SEM sample holder, then coated with osmium. Gas adsorption measurements were performed using MicrotracBEL model BELSORP-mini. Particle size distribution was analyzed using HORIBA model laser scattering particle size analyzer Partica LA-950. The samples were dispersed in acetone at 25 °C. X-ray fluorescence (XRF) data were recorded on a Rigaku model Energy Dispersive X-ray Fluorescence Spectrometer EDXL 300LC using Mo as a secondary target. Confocal laser scanning microscope (CLSM) measurements were performed on an Evident Fluoview FV4000 using 405 nm violet light as an excitation light.

2. Materials

All reagents and chemicals used in this study were obtained from Merck KGaA, FUJIFILM Wako Pure Chemicals, and Tokyo Chemical Industry, unless otherwise noted. Deuterated solvents for NMR spectroscopy were purchased from Cambridge Isotope Laboratories.

3. Synthesis of MOF Stationary Phases

Synthesis of Zn-BDC

Zn-BDC ($[Zn_2(bdc)_2ted]_n$) (bdc = 1,4-benzenedicarboxylate, ted = triethylenediamine) was synthesized according to a previously reported procedure with slight modifications.[1–4] $Zn(NO_3)_2·6H_2O$ (4.46 g, 15 mmol) and H_2bdc (2.49 g, 15 mmol) were dissolved in *N*,*N*-dimethylformamide (DMF, 150 mL). A separate DMF solution (150 mL) of ted (1.68 g, 15 mmol) was then added to the mixture. The resulting solution was stirred vigorously at 120 °C for 48 h. After cooling to room temperature, the resulting solid was collected by suction filtration and washed with fresh DMF (approximately 100 mL) on the filter. The obtained powdery microcrystals, containing DMF as the guest molecule, were evacuated under reduced pressure at 120 °C for 16 h to afford activated **Zn-BDC** as a bulk powder (4.07 g).

Synthesis of Zn-NDC

Zn-NDC ($[Zn_2(ndc)_2ted]_n$) (ndc = 1,4-naphthalenedicarboxylate) was synthesized according to a previously reported procedure with slight modifications.[2–5] $Zn(NO_3)_2·6H_2O$ (4.46 g, 15 mmol) and H_2ndc (3.24 g, 15 mmol) were dissolved in DMF (150 mL). A separate DMF solution (150 mL) of ted (1.68 g, 15 mmol) was then added to the mixture. The resulting

solution was stirred vigorously at 120 °C for 48 h. After cooling to room temperature, the resulting solid was collected by suction filtration and washed with fresh DMF (approximately 100 mL) on the filter. The obtained powdery microcrystals, containing DMF as the guest molecule, were evacuated under reduced pressure at 120 °C for 16 h to afford activated **Zn-NDC** as a bulk powder (4.71 g).

Synthesis of Cu-BDC

Cu-BDC ($[Cu_2(bdc)_2ted]_n$) (bdc = 1,4-benzenedicarboxylate, ted = triethylenediamine) was synthesized in analogy to the procedure for the synthesis of **Zn-BDC** and **Zn-NDC**. $Cu(NO_3)_2 \cdot 2.5H_2O$ (1.40 g, 6.0 mmol) and H_2bdc (0.997 g, 6.0 mmol) were dissolved in DMF (60 mL). A separate DMF solution (60 mL) of ted (0.673 g, 6.0 mmol) was then added to the mixture. The resulting solution was stirred vigorously at 120 °C for 48 h. After cooling to room temperature, the resulting solid was collected by suction filtration and washed with fresh DMF (approximately 100 mL) on the filter. The obtained powdery microcrystals, containing DMF as the guest molecule, were evacuated under reduced pressure at 120 °C for 16 h to afford activated **Cu-BDC** as a bulk powder (1.37 g).

Synthesis of Cu-NDC

Cu-NDC ($[Cu_2(ndc)_2ted]_n$) (bdc = 1,4-benzenedicarboxylate, ted = triethylenediamine) was synthesized in analogy to the procedure for the synthesis of **Zn-BDC** and **Zn-NDC**. $Cu(NO_3)_2 \cdot 2.5H_2O$ (1.40 g, 6.0 mmol) and H_2ndc (1.30 g, 6.0 mmol) were dissolved in DMF (60 mL). A separate DMF solution (60 mL) of ted (0.673 g, 6.0 mmol) was then added to the mixture. The resulting solution was stirred vigorously at 120 °C for 48 h. After cooling to room temperature, the resulting solid was collected by suction filtration and washed with fresh DMF (approximately 100 mL) on the filter. The obtained powdery microcrystals, containing DMF as the guest molecule, were evacuated under reduced pressure at 120 °C for 16 h to afford activated **Cu-NDC** as a bulk powder (1.83 g).

Solvothermal Synthesis of Cu-NDC-on-Zn-BDC

$Cu(NO_3)_2 \cdot 2.5H_2O$ (0.698 g, 3.0 mmol) and H_2ndc (0.649 g, 3.0 mmol) were dissolved in DMF (30 mL). A separate DMF solution (30 mL) of ted (0.336 g, 3.0 mmol) was then added to the mixture. The resulting solution was centrifuged, and 40 mL of the supernatant solution was added to a separate DMF suspension (30 mL) of pre-synthesized **Zn-BDC** microcrystals (500 mg). The resulting mixture was stirred vigorously at 120 °C for 48 h. After cooling to room temperature, the resulting green solid was collected by suction filtration and washed with fresh DMF (approximately 100 mL) on the filter. The obtained powdery microcrystals, containing DMF as the guest molecule, were evacuated under reduced pressure at 120 °C for 16 h to afford activated **Cu-NDC-on-Zn-BDC** as a bulk powder (0.688 g).

Layer-by-Layer Synthesis of Cu-NDC-on-Zn-BDC

Cu-NDC-on-Zn-BDC was synthesized through a layer-by-layer (LbL) method.[6,7] **Zn-BDC** (500 mg) was dispersed in 40 mL of a DMF solution containing $Cu(NO_3)_2{\cdot}2.5H_2O$ at a concentration of 1.25 mM (or 2.5 mM or 12.5 mM when specified). The mixture was stirred vigorously at 120 °C for 15 min, then cooled to room temperature. The solid was collected by centrifugation and washed three times with fresh DMF (approximately 20 mL per wash) using centrifugation and decantation. The recovered solid was then dispersed in 40 mL of a DMF solution containing H_2ndc and ted, each at a concentration of 1.25 mM (or 2.5 mM or 12.5 mM when specified). The suspension was stirred at 120 °C for 30 min, cooled to room temperature, and collected by centrifugation. The solid was again washed three times with fresh DMF as described above. This two-step cycle of metal and ligand treatment was repeated five times (or one or three times when indicated). The resulting green powder was evacuated under reduced pressure at 120 °C for 16 h to yield the bulk sample of **Cu-NDC-on-Zn-BDC** (0.525 g).

Layer-by-Layer Synthesis of Cu-BDC-on-Zn-NDC

Cu-BDC-on-Zn-NDC was synthesized through a layer-by-layer method.[6,7] **Zn-NDC** (500 mg) was dispersed in 40 mL of a DMF solution containing $Cu(NO_3)_2{\cdot}2.5H_2O$ at a concentration of 1.25 mM (or 2.5 mM or 12.5 mM when specified). The mixture was stirred vigorously at 120 °C for 15 min, then cooled to room temperature. The solid was collected by centrifugation and washed three times with fresh DMF (approximately 20 mL per wash) using centrifugation and decantation. The recovered solid was then dispersed in 40 mL of a DMF solution containing H_2bdc and ted, each at a concentration of 1.25 mM (or 2.5 mM or 12.5 mM when specified). The suspension was stirred at 120 °C for 30 min, cooled to room temperature, and collected by centrifugation. The solid was again washed three times with fresh DMF as described above. This two-step cycle of metal and ligand treatment was repeated five times (or one or three times when indicated). The resulting bluish powder was evacuated under reduced pressure at 120 °C for 16 h to yield the bulk sample of **Cu-BDC-on-Zn-NDC** (0.539 g).

Layer-by-Layer Synthesis of Zn-NDC-on-Zn-BDC

Zn-NDC-on-Zn-BDC was synthesized through a layer-by-layer method.[6,7] **Zn-BDC** (500 mg) was dispersed in 40 mL of a DMF solution containing $Zn(NO_3)_2{\cdot}6H_2O$ at a concentration of 2.5 mM (or 12.5 mM when specified). The mixture was stirred vigorously at 120 °C for 15 min, then cooled to room temperature. The solid was collected by centrifugation and washed three times with fresh DMF (approximately 20 mL per wash) using centrifugation and decantation. The recovered solid was then dispersed in 40 mL of a DMF solution containing H_2ndc and ted, each at a concentration of 2.5 mM (or 12.5 mM when specified). The suspension was stirred at 120 °C for 30 min, cooled to room temperature, and collected by centrifugation.

The solid was again washed three times with fresh DMF as described above. This two-step cycle of metal and ligand treatment was repeated five times. The resulting white powder was evacuated under reduced pressure at 120 °C for 16 h to yield the bulk sample of **Zn-NDC-on-Zn-BDC** (0.470 g).

Layer-by-Layer Synthesis of Zn-BDC-on-Zn-NDC

Zn-BDC-on-Zn-NDC was synthesized through a layer-by-layer method.[6,7] **Zn-NDC** (500 mg) was dispersed in 40 mL of a DMF solution containing $Zn(NO_3)_2 \cdot 6H_2O$ at a concentration of 2.5 mM (or 12.5 mM when specified). The mixture was stirred vigorously at 120 °C for 15 min, then cooled to room temperature. The solid was collected by centrifugation and washed three times with fresh DMF (approximately 20 mL per wash) using centrifugation and decantation. The recovered solid was then dispersed in 40 mL of a DMF solution containing H_2bdc and ted, each at a concentration of 2.5 mM (or 12.5 mM when specified). The suspension was stirred at 120 °C for 30 min, cooled to room temperature, and collected by centrifugation. The solid was again washed three times with fresh DMF as described above. This two-step cycle of metal and ligand treatment was repeated five times. The resulting white powder was evacuated under reduced pressure at 120 °C for 16 h to yield the bulk sample of **Zn-BDC-on-Zn-NDC** (0.527 g).

Synthesis of Solid Solution MOF

Solid solution MOF was synthesized according to the previously reported procedure with slight modifications.[4] $Zn(NO_3)_2 \cdot 6H_2O$ (0.892 g, 3.0 mmol), $Cu(NO_3)_2 \cdot 2.5H_2O$, H_2bdc (0.498 g, 3.0 mmol), and H_2ndc (0.065 g, 0.30 mmol) were dissolved in DMF (30 mL). A separate DMF solution (30 mL) of ted (0.370 g, 3.3 mmol) was then added to the mixture. The resulting solution was stirred vigorously at 120 °C for 48 h. After cooling to room temperature, the resulting solid was collected by suction filtration and washed with fresh DMF (approximately 100 mL) on the filter. The obtained powdery microcrystals, containing DMF as the guest molecule, were evacuated under reduced pressure at 120 °C for 16 h to afford activated solid-solution MOF as a bulk powder (1.05 g).

4. Batch Adsorption Experiments

Batch adsorption experiments were conducted using 10 mg of MOF in 10 mL of a perylene solution (initial concentration: 1.5 mg mL^{-1}). The concentration of perylene in the supernatant was monitored over time by UV–vis spectroscopy to calculate the amount adsorbed. Toluene was selected as the solvent for these experiments due to its high solubility for perylene, which enabled adsorption studies at higher concentrations and provided reliable measurements of both adsorption rates and capacities. In contrast, hexane, which was used in the LC experiments, has a lower solubility for perylene and a lower boiling point, which prevented accurate

quantification in batch adsorption studies. Therefore, toluene was used for the adsorption experiments, while hexane remained the solvent of choice for LC evaluations due to its compatibility with common LC protocols.

5. Supporting Tables

Table S1. Particle size of the MOF-on-MOF and their parent core MOF microcrystals.[a]

core MOF	Particle size (μm)	MOF-on-MOF (shell MOF fraction)	Particle size (μm)
**Zn-BDC**	8.2 ± 3.1	**Cu-NDC-on-Zn-BDC** (0.10)	8.3 ± 2.7
**Zn-BDC**	10.5 ± 3.6	**Cu-NDC-on-Zn-BDC** (0.15)	11.5 ± 3.8
**Zn-BDC**	10.0 ± 3.9	**Cu-NDC-on-Zn-BDC** (0.26)	10.4 ± 3.1
**Zn-NDC**	3.5 ± 1.5	**Cu-BDC-on-Zn-NDC** (0.10)	2.4 ± 1.2
**Zn-NDC**	3.5 ± 1.5	**Cu-BDC-on-Zn-NDC** (0.21)	2.4 ± 1.2
**Zn-NDC**	3.5 ± 1.5	**Cu-BDC-on-Zn-NDC** (0.53)	3.3 ± 0.8

[a]Determined by the particle size analyser for the samples dispersed in acetone at room temperature.

Table S2. x_{NMR} and x_{XRF} values for the MOF-on-MOF microcrystals.

MOF-on-MOF (shell MOF fraction)	x_{NMR}[a]	x_{XRF}[b]
**Cu-NDC-on-Zn-BDC** (0.10)	0.10	0.12
**Cu-NDC-on-Zn-BDC** (0.15)	0.15	0.15
**Cu-NDC-on-Zn-BDC** (0.26)	0.26	0.38
**Cu-BDC-on-Zn-NDC** (0.10)	0.10	0.11
**Cu-BDC-on-Zn-NDC** (0.21)	0.21	0.23
**Cu-BDC-on-Zn-NDC** (0.53)	0.53	0.36

[a]Fraction of the shell ligand (either bdc or ndc) among the total ligands (both bdc and ndc) constituting the MOF particles, determined by ^{1}H NMR measurements.
[b]Fraction of the shell metal ion (either Cu^{2+} or Zn^{2+}) among the total metal ions (both Cu^{2+} and Zn^{2+}) constituting the MOF particles, determined by XRF measurements.

Table S3. Retention factor (k) of anthracene, perylene, and phenanthrene, measured on the **Cu-NDC-on-Zn-BDC** stationary phases.[a]

Stationary phase (shell fraction, x_{NMR})	anthracene	perylene	phenanthrene
**Zn-BDC** (x_{NMR} = 0)	5.56	11.5	2.12
**Cu-NDC-on-Zn-BDC** (x_{NMR} = 0.10)	6.41	3.86	N.D.[b]
**Cu-NDC-on-Zn-BDC** (x_{NMR} = 0.15)	8.43	3.73	N.D.[b]
**Cu-NDC-on-Zn-BDC** (x_{NMR} = 0.26)	6.63	2.19	N.D.[b]
**Cu-NDC** (x_{NMR} = 1)	5.33	1.42	N.D.[b]

[a]LC condition: eluent, hexane; temperature, 40 °C; flow rate, 1.0 ml min^{-1}; detector: ELSD.
[b]The analyte did not elute from the column due to the strong adsorption (Figure S29).

Table S4. The retention factor ratio (A) calculated for perylene and phenanthrene on the **Cu-NDC-on-Zn-BDC** stationary phases.[a]

Stationary phase (shell fraction, x_{NMR})	perylene	phenanthrene
**Zn-BDC** (x_{NMR} = 0)	2.06	0.38
**Cu-NDC-on-Zn-BDC** (x_{NMR} = 0.10)	0.60	N.D.[b]
**Cu-NDC-on-Zn-BDC** (x_{NMR} = 0.15)	0.44	N.D.[b]
**Cu-NDC-on-Zn-BDC** (x_{NMR} = 0.26)	0.32	N.D.[b]
**Cu-NDC** (x_{NMR} = 1)	0.27	N.D.[b]

[a]A is the retention factor ratio defined as $A = k/k_{ant}$, where k is the retention factor of the analyte and k_{ant} is that of anthracene, used as a standard. The k values are shown in Table S3.
[b]The analyte did not elute from the column due to the strong adsorption (Figure S29).

Table S5. Retention factor (k) of anthracene, perylene, and phenanthrene, measured on the **Cu-BDC-on-Zn-NDC** stationary phases.[a]

Stationary phase (shell fraction, x_{NMR})	anthracene	perylene	phenanthrene
**Zn-NDC** (x_{NMR} = 0)	2.90	0.86	3.60
**Cu-BDC-on-Zn-NDC** (x_{NMR} = 0.10)	4.59	2.04	5.35
**Cu-BDC-on-Zn-NDC** (x_{NMR} = 0.21)	5.95	3.41	6.84
**Cu-BDC-on-Zn-NDC** (x_{NMR} = 0.53)	6.83	6.51	6.85
**Cu-BDC** (x_{NMR} = 1)	8.48	12.5	7.68

[a]LC condition: eluent, hexane; temperature, 40 °C; flow rate, 1.0 ml min^{-1}; detector: ELSD.

Table S6. The retention factor ratio (A) calculated for perylene and phenanthrene on the **Cu-BDC-on-Zn-NDC** stationary phases.[a]

Stationary phase (shell fraction, x_{NMR})	perylene	phenanthrene
**Zn-NDC** (x_{NMR} = 0)	0.30	1.24
**Cu-BDC-on-Zn-NDC** (x_{NMR} = 0.10)	0.44	1.17
**Cu-BDC-on-Zn-NDC** (x_{NMR} = 0.21)	0.57	1.15
**Cu-BDC-on-Zn-NDC** (x_{NMR} = 0.53)	0.95	1.00
**Cu-BDC** (x_{NMR} = 1)	1.47	0.91

[a]A is the retention factor ratio defined as $A = k/k_{ant}$, where k is the retention factor of the analyte and k_{ant} is that of anthracene, used as a standard. The k values are shown in Table S5.

Table S7. Retention factor (k) of anthracene, perylene, and phenanthrene, measured on the **Zn-NDC-on-Zn-BDC** stationary phases.[a]

Stationary phase (shell fraction, x_{NMR})	anthracene	perylene	phenanthrene
**Zn-BDC** (x_{NMR} = 0)	5.56	11.5	2.12
**Zn-NDC-on-Zn-BDC** (x_{NMR} = 0.11)	4.69	2.25	4.36
**Zn-NDC-on-Zn-BDC** (x_{NMR} = 0.29)	4.43	1.71	4.47
**Zn-NDC** (x_{NMR} = 1)	2.90	0.86	3.60

[a]LC condition: eluent, hexane; temperature, 40 °C; flow rate, 1.0 ml min^{-1}; detector: ELSD.

Table S8. The retention factor ratio (A) calculated for perylene and phenanthrene on the **Zn-NDC-on-Zn-BDC** stationary phases.[a]

Stationary phase (shell fraction, x_{NMR})	perylene	phenanthrene
**Zn-BDC** (x_{NMR} = 0)	2.06	0.38
**Zn-NDC-on-Zn-BDC** (x_{NMR} = 0.11)	0.48	0.93
**Zn-NDC-on-Zn-BDC** (x_{NMR} = 0.29)	0.38	1.01
**Zn-NDC** (x_{NMR} = 1)	0.30	1.24

[a]A is the retention factor ratio defined as $A = k/k_{ant}$, where k is the retention factor of the analyte and k_{ant} is that of anthracene, used as a standard. The k values are shown in Table S7.

Table S9. Retention factor (k) of anthracene, perylene, and phenanthrene, measured on the **Zn-BDC-on-Zn-NDC** stationary phases.[a]

Stationary phase (shell fraction, x_{NMR})	anthracene	perylene	phenanthrene
**Zn-NDC** (x_{NMR} = 0)	2.90	0.86	3.60
**Zn-BDC-on-Zn-NDC** (x_{NMR} = 0.16)	3.80	2.70	3.49
**Zn-BDC-on-Zn-NDC** (x_{NMR} = 0.71)	8.29	10.7	6.46
**Zn-BDC** (x_{NMR} = 1)	5.56	11.5	2.12

[a]LC condition: eluent, hexane; temperature, 40 °C; flow rate, 1.0 ml min^{-1}; detector: ELSD.

Table S10. The retention factor ratio (A) calculated for perylene and phenanthrene on the **Zn-BDC-on-Zn-NDC** stationary phases.[a]

Stationary phase (shell fraction, x_{NMR})	perylene	phenanthrene
**Zn-NDC** (x_{NMR} = 0)	0.30	1.24
**Zn-BDC-on-Zn-NDC** (x_{NMR} = 0.16)	0.71	0.92
**Zn-BDC-on-Zn-NDC** (x_{NMR} = 0.71)	1.29	0.77
**Zn-BDC** (x_{NMR} = 1)	2.06	0.38

[a]A is the retention factor ratio defined as $A = k/k_{ant}$, where k is the retention factor of the analyte and k_{ant} is that of anthracene, used as a standard. The k values are shown in Table S9.

6. Supporting Figures

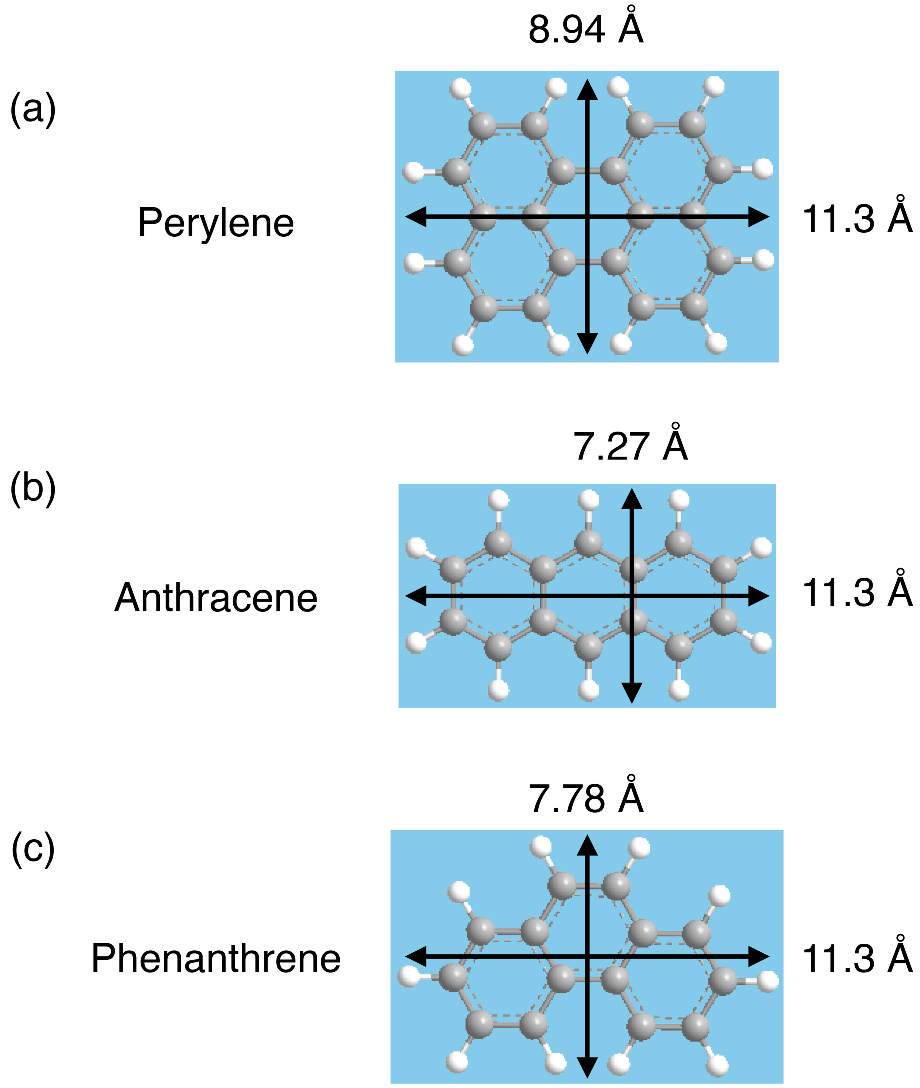

Figure S1. Molecular dimensions of PAHs used as the model analyte in this study: (a) perylene, (b) anthracene, and (c) phenanthrene.

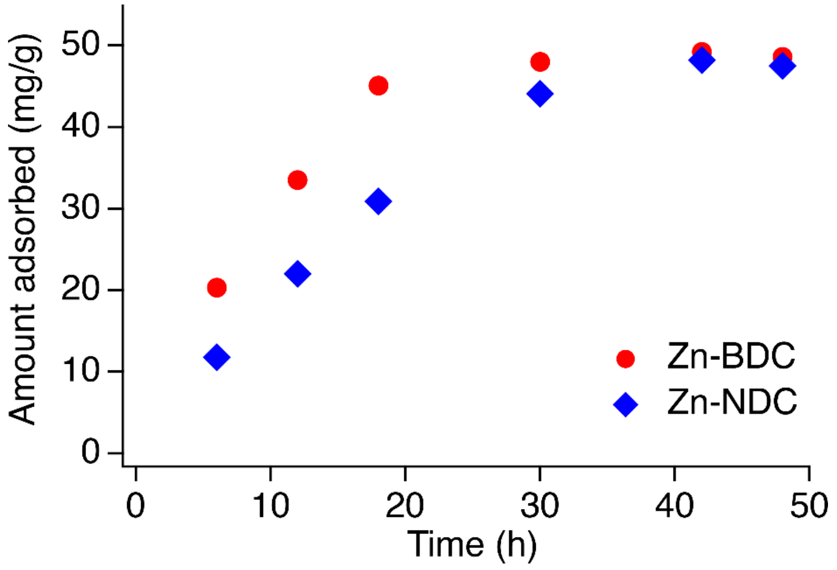

Figure S2. Results of batch adsorption experiments for perylene using **Zn-BDC** (red circles) and **Zn-NDC** (blue rhombi), performed in toluene at 40 °C (see Section 4 for the experimental details). Both MOFs exhibited similar adsorption capacities (~50 mg g^{-1}) under these conditions; however, **Zn-BDC** showed faster adsorption kinetics than **Zn-NDC**. This difference is attributed to the larger pore size of **Zn-BDC**, which facilitates more rapid diffusion of perylene compared to the smaller pores of **Zn-NDC**.

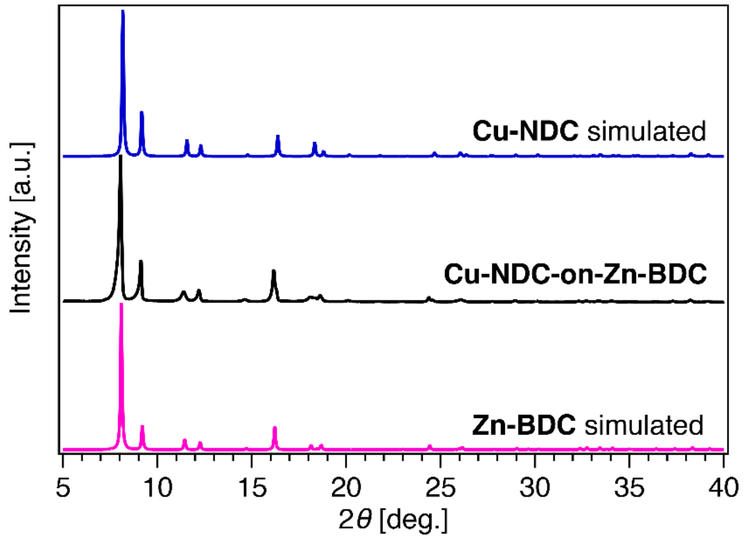

Figure S3. PXRD data of **Cu-NDC-on-Zn-BDC** synthesized through a conventional solvothermal method (black trace). Simulated PXRD patterns of pure **Zn-BDC** and **Cu-NDC** are shown as magenta and blue traces, respectively.

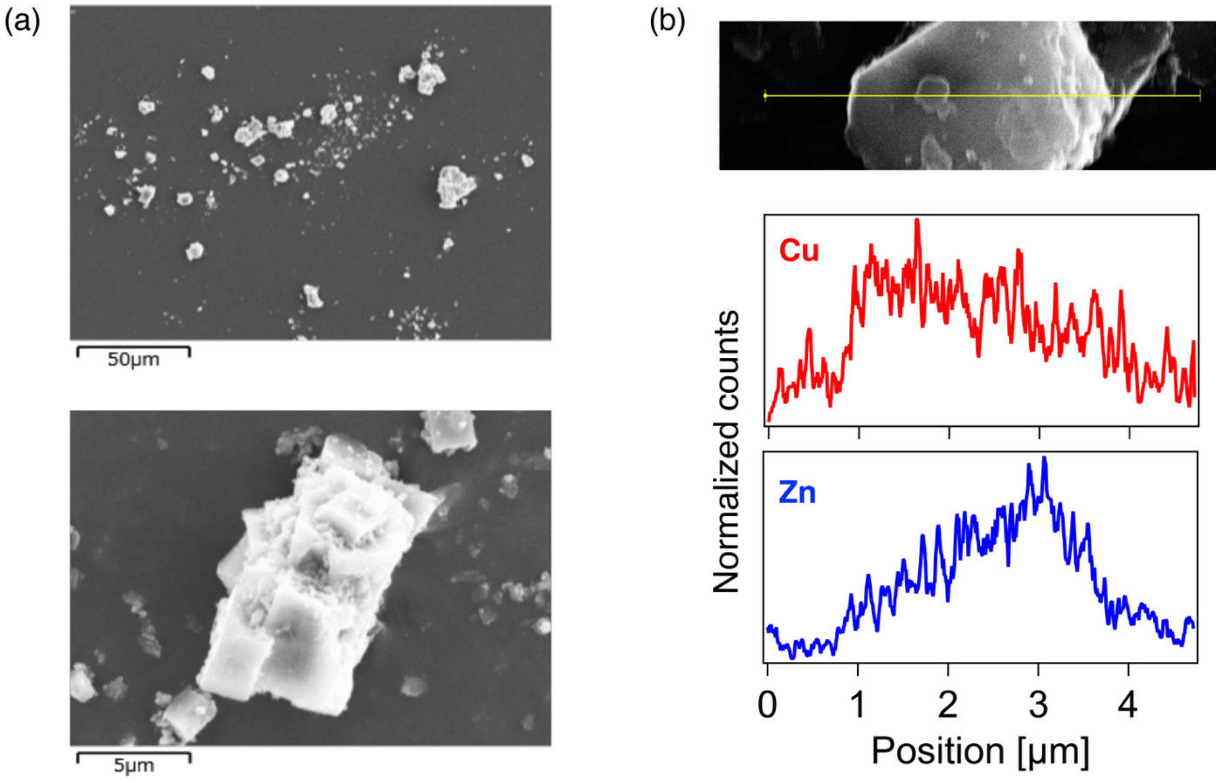

Figure S4. SEM-EDX analysis of **Cu-NDC-on-Zn-BDC** synthesized via a conventional solvothermal method. (a) Representative SEM images of **Cu-NDC-on-Zn-BDC** particles. (b) EDX line-scan profile (bottom) showing the distribution of Cu (red) and Zn (blue) along the yellow line indicated in the corresponding SEM image (top).

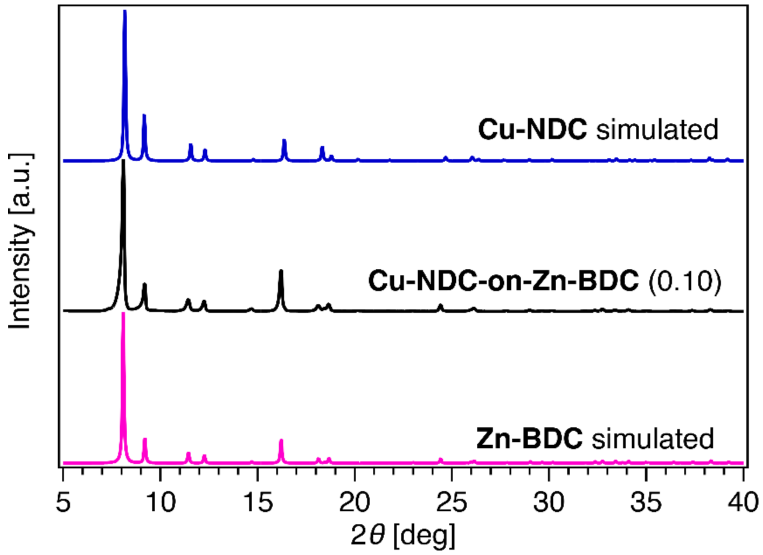

Figure S5. PXRD data of **Cu-NDC-on-Zn-BDC** (shell MOF fraction $x_{NMR} = 0.10$) synthesized through the layer-by-layer (LbL) method (black trace). Simulated PXRD patterns of pure **Zn-BDC** and **Cu-NDC** are shown as magenta and blue traces, respectively.

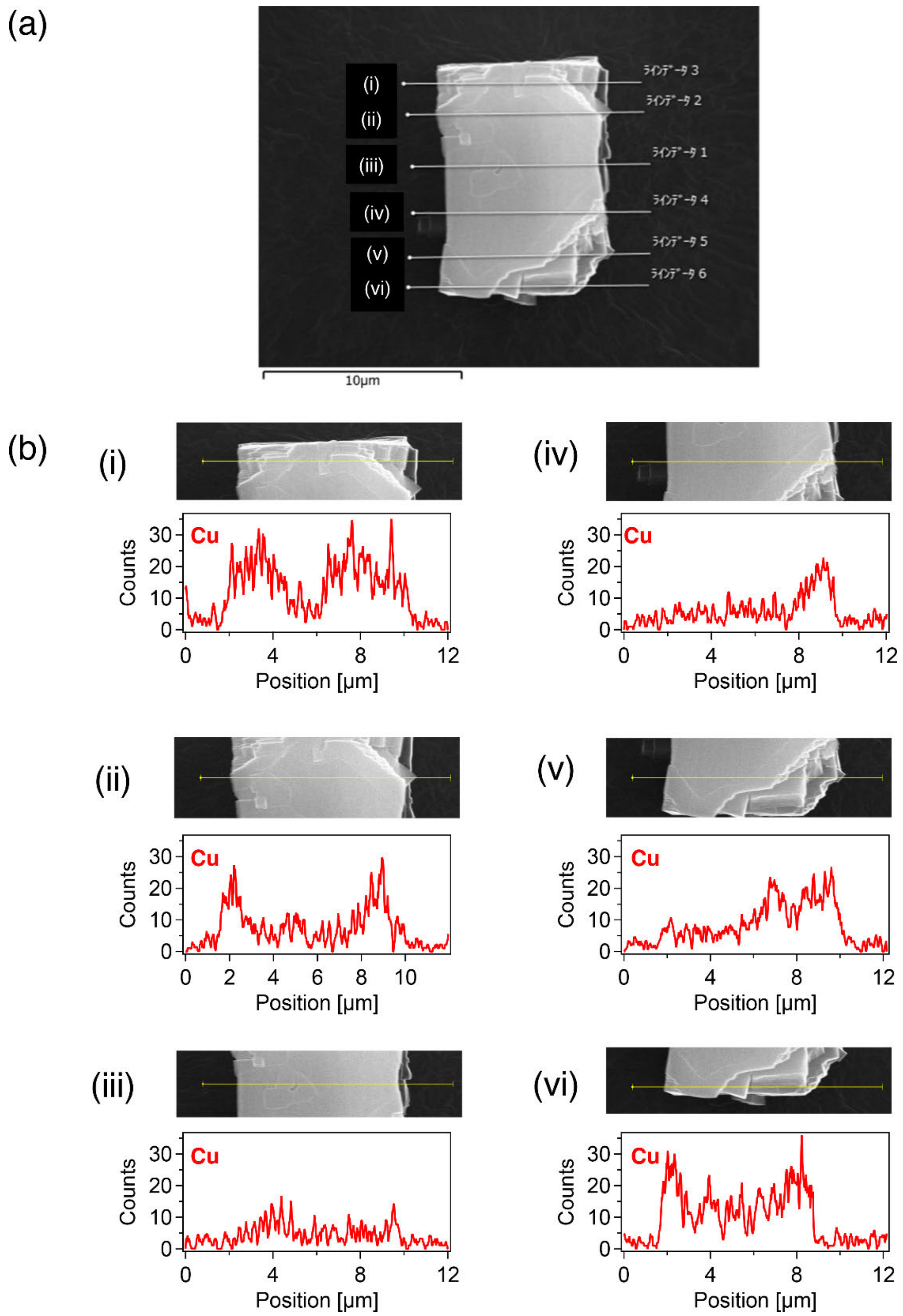

Figure S6. (a) A SEM image of a representative crystal of **Cu-NDC-on-Zn-BDC** ($x_{\mathrm{NMR}} = 0.10$). (b) EDX line-scan profiles showing the distribution of Cu along the yellow lines (i-vi) indicated in panel (a).

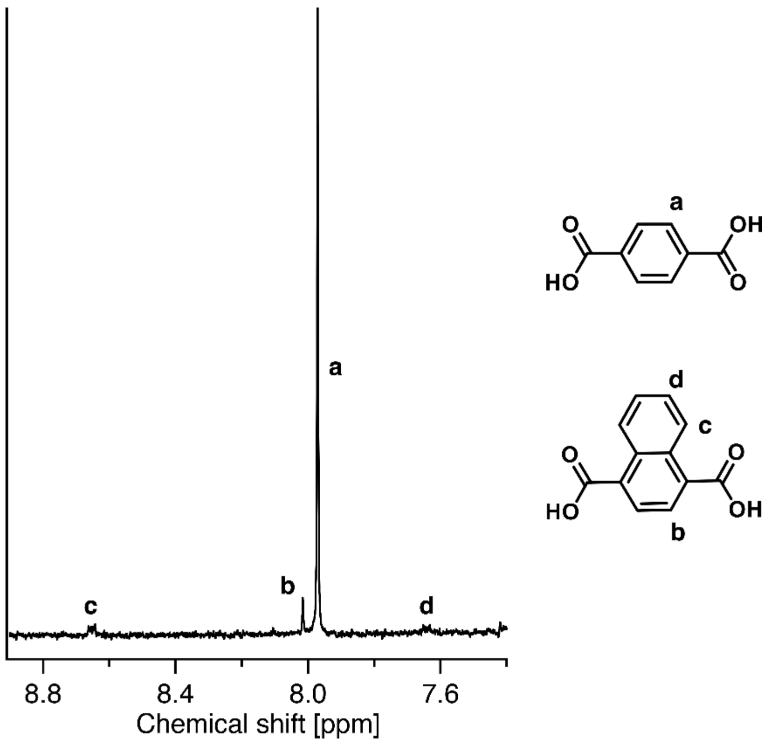

Figure S7. ^{1}H NMR spectrum of aromatic protons for **Cu-NDC-on-Zn-BDC** (x_{NMR} = 0.10), measured after digestion. The sample was digested in DMSO-d_6/DCl (9/1, v/v) mixture and subsequently subjected to the NMR measurement.

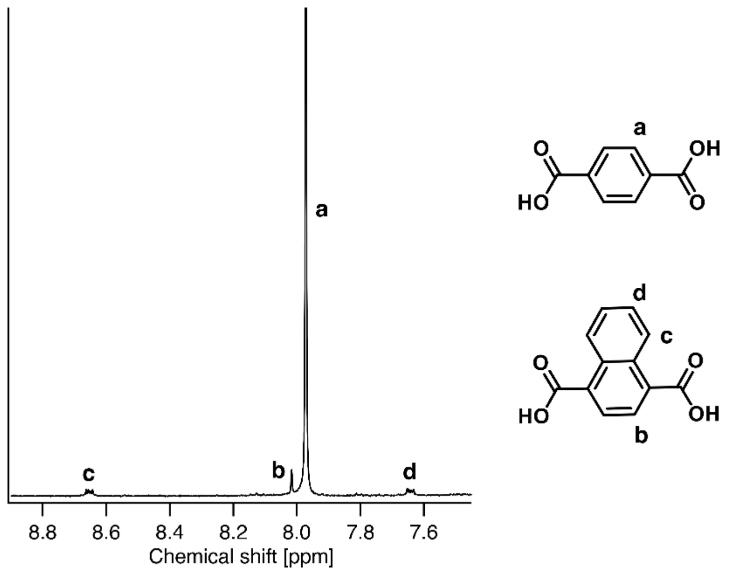

Figure S8. ^{1}H NMR spectrum of aromatic protons for solid-solution MOF with the ndc fraction of 0.09, measured after digestion. The sample was digested in DMSO-d_6/DCl (9/1, v/v) mixture and subsequently subjected to the NMR measurement.

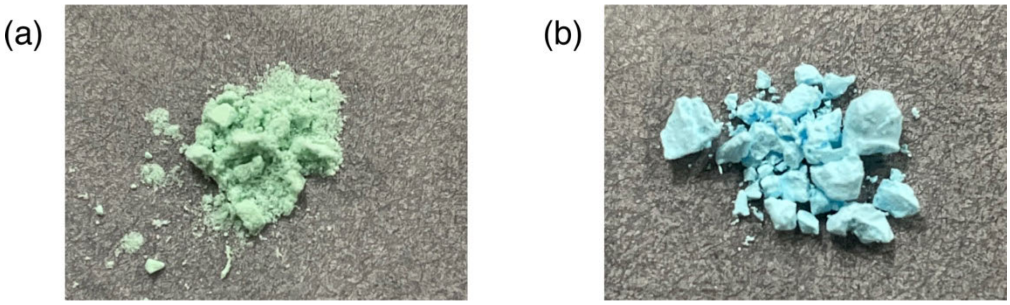

Figure S9. Digital photographs of (a) **Cu-NDC-on-Zn-BDC** (x_{NMR} = 0.10) and (b) solid solution MOF (ndc fraction = 0.09) samples.

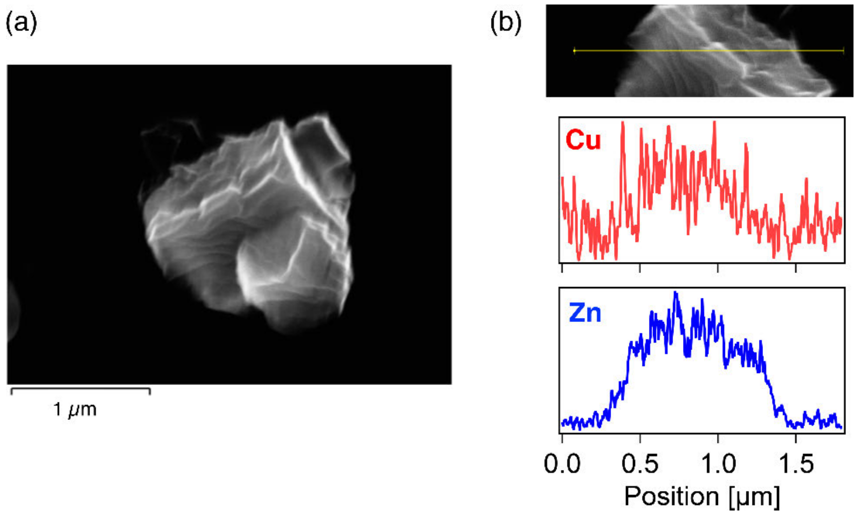

Figure S10. (a) A SEM image of a representative crystal of the solid-solution MOF (ndc fraction = 0.09). (b) EDX line-scan profile (bottom) showing the distribution of Cu (red) and Zn (blue) along the yellow line indicated in the corresponding SEM image (top).

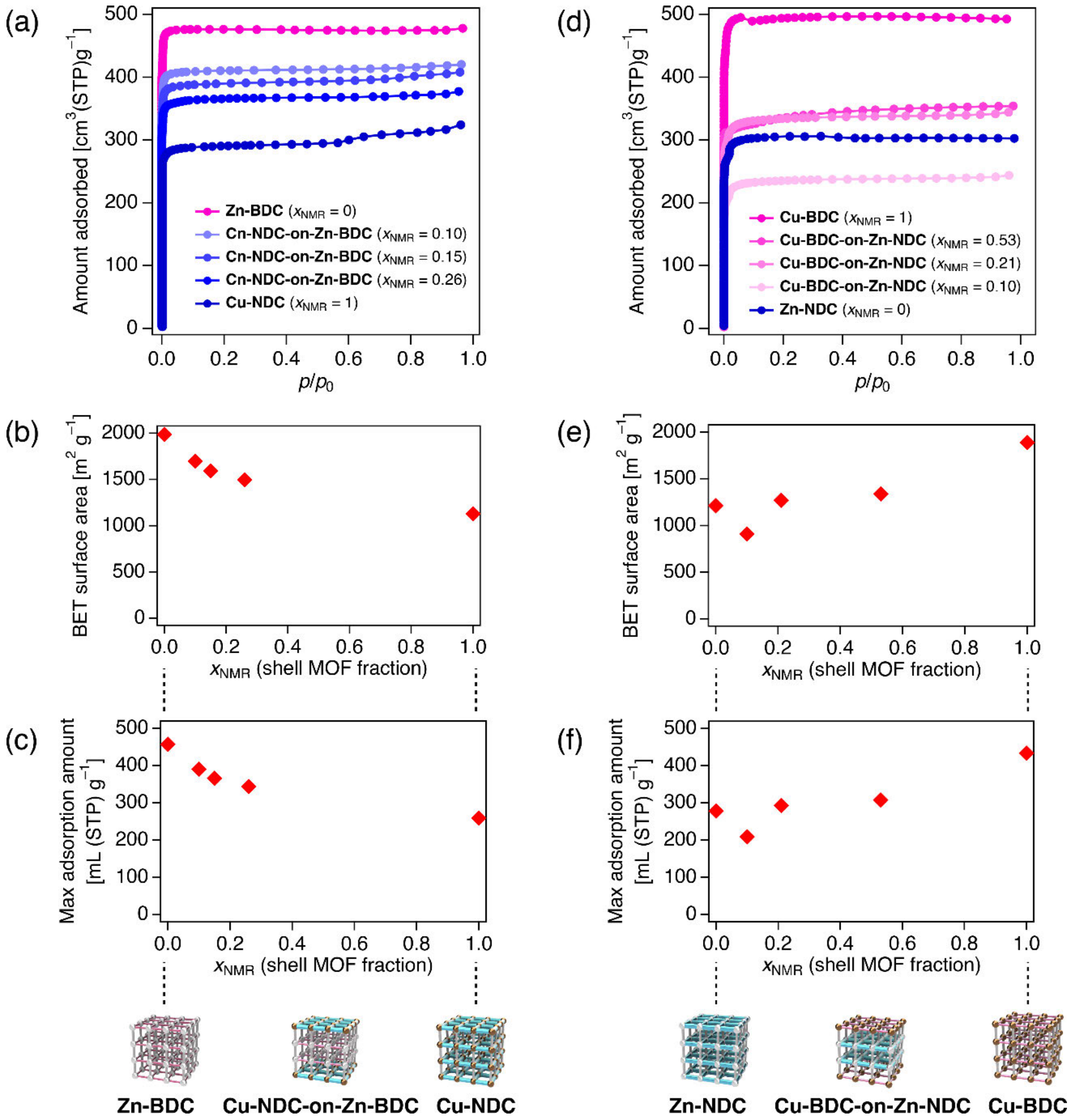

Figure S11. (a–c) Results of N_2 adsorption measurements for the **Cu-NDC-on-Zn-BDC** samples with varying shell MOF fractions: (a) adsorption isotherms measured at 77 K, (b) BET surface area, and (c) maximum adsorption amount at $p/p_0 = 0.99$. The shell MOF fractions of $x_{NMR} = 0$ and 1 correspond to the parent MOFs **Zn-BDC** and **Cu-NDC**, respectively. (d–f) Results of N_2 adsorption measurements for the **Cu-BDC-on-Zn-NDC** samples with varying shell MOF fractions: (d) adsorption isotherms measured at 77 K, (e) BET surface area, and (f) maximum adsorption amount at $p/p_0 = 0.99$. The shell MOF fractions of $x_{NMR} = 0$ and 1 correspond to the parent MOFs **Zn-NDC** and **Cu-BDC**, respectively. The relatively large variation in BET surface area and maximum adsorption amount observed for the **Cu-BDC-on-Zn-NDC** series is likely due to the moisture sensitivity of the outermost **Cu-BDC** layer. Because exposure to air is unavoidable during sample preparation and measurement, this layer may undergo partial deterioration, which would reduce the gas uptake relative to that expected from the BDC shell content.

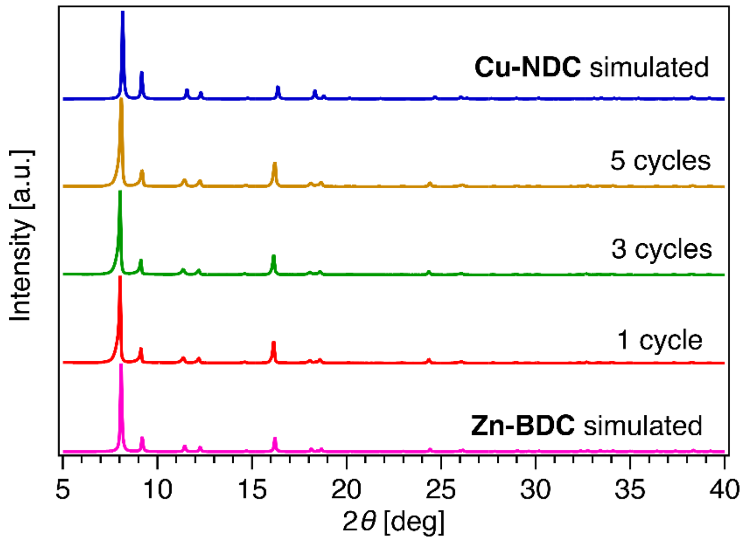

Figure S12. PXRD data of **Cu-NDC-on-Zn-BDC** synthesized through 1 (red), 3 (green), and 5 (khaki) LbL cycles. Simulated PXRD patterns of pure **Zn-BDC** and **Cu-NDC** are shown as magenta and blue traces, respectively.

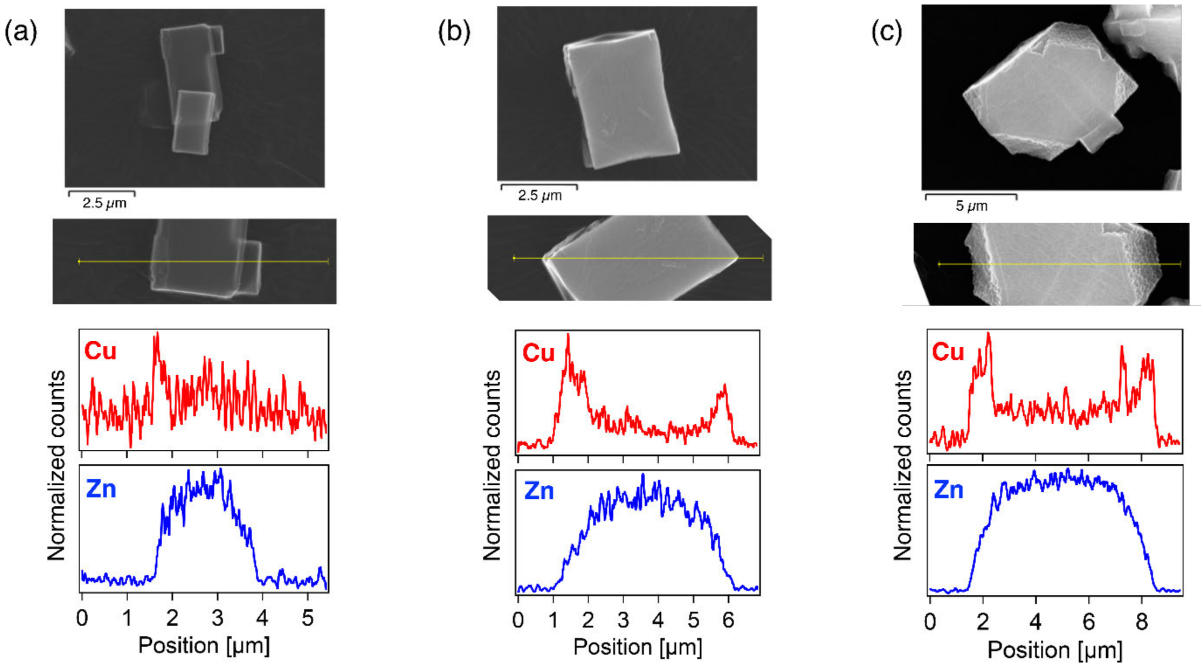

Figure S13. (a–c) SEM images (top, middle) and EDX line-scanning profiles (bottom) of **Cu-NDC-on-Zn-BDC** microcrystals synthesized with (a) 1, (b) 3, and (c) 5 LbL cycles. EDX line-scan profiles show the distribution of Cu (red) and Zn (blue) along the yellow line indicated in the corresponding SEM image (middle).

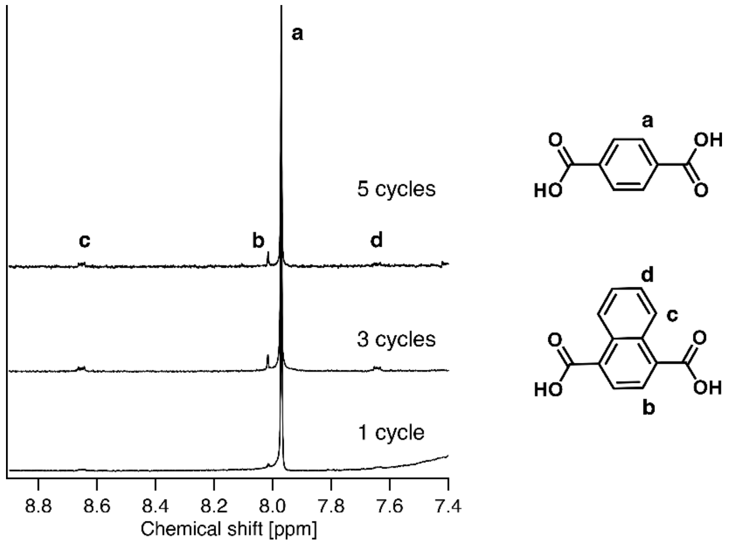

Figure S14. ^{1}H NMR spectra of aromatic protons for **Cu-NDC-on-Zn-BDC** samples synthesized with different number of LbL cycles, measured after digestion. The samples were digested in DMSO-d_6/DCl (9/1, v/v) mixture and subsequently subjected to the NMR measurement.

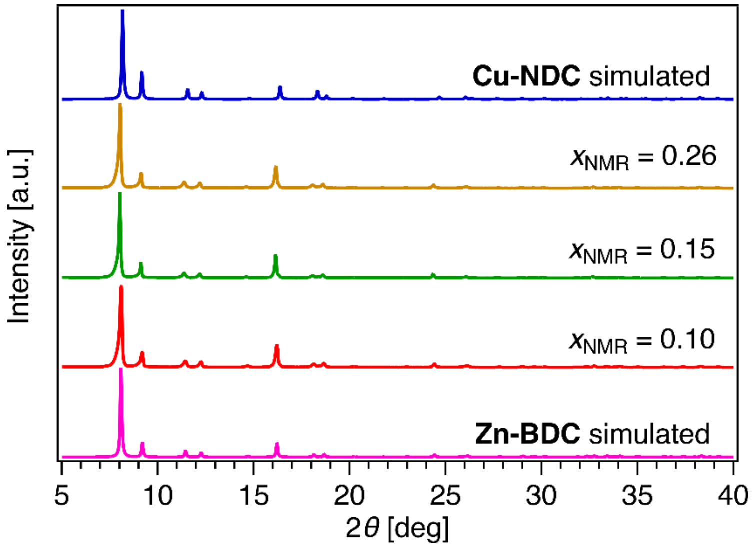

Figure S15. PXRD data of **Cu-NDC-on-Zn-BDC** with varying shell fractions: x_{NMR} = 0.10 (red), x_{NMR} = 0.15 (green), and x_{NMR} = 0.26 (khaki). Simulated PXRD patterns of pure **Zn-BDC** and **Cu-NDC** are shown as magenta and blue traces, respectively.

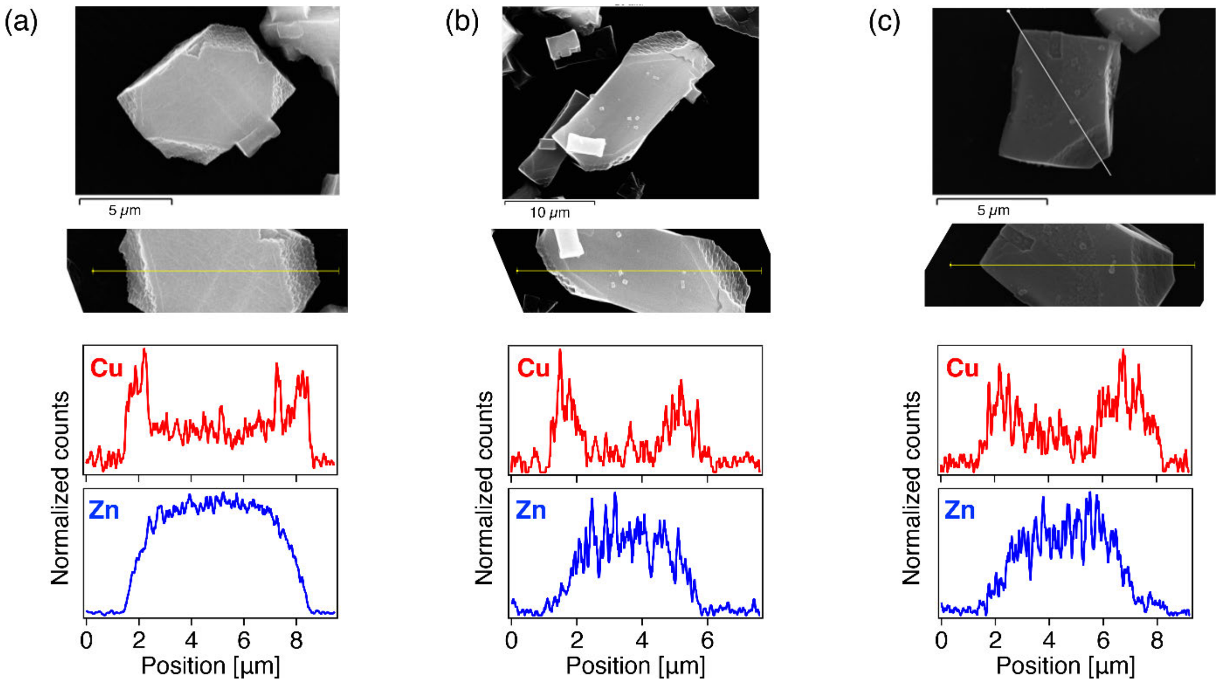

Figure 16. (a–c) SEM images (top, middle) and EDX line-scanning profiles (bottom) of **Cu-NDC-on-Zn-BDC** microcrystals with varying shell fractions: (a) x_{NMR} = 0.10, (b) x_{NMR} = 0.15, and (c) x_{NMR} = 0.26. EDX line-scan profiles show the distribution of Cu (red) and Zn (blue) along the yellow line indicated in the corresponding SEM image (middle).

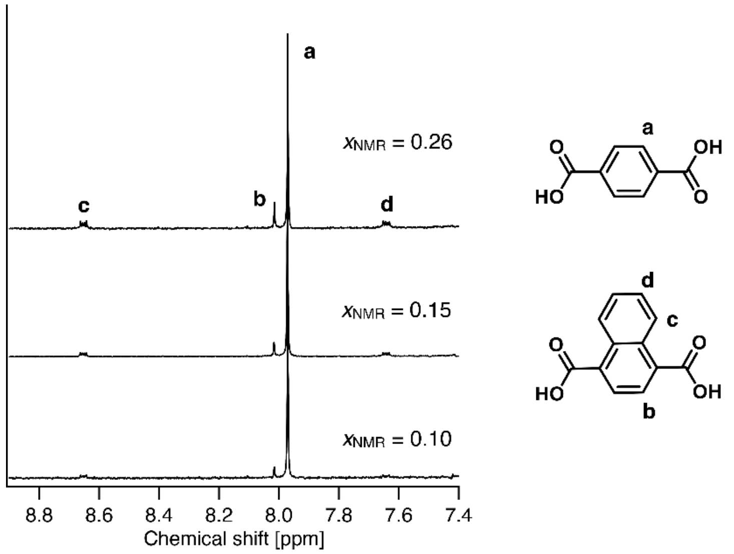

Figure S17. ^{1}H NMR spectra of aromatic protons for **Cu-NDC-on-Zn-BDC** samples with varying shell fractions: x_{NMR} = 0.10 (bottom), x_{NMR} = 0.15 (middle), and x_{NMR} = 0.26 (top), measured after digestion. The samples were digested in DMSO-d_6/DCl (9/1, v/v) mixture and subsequently subjected to the NMR measurement.

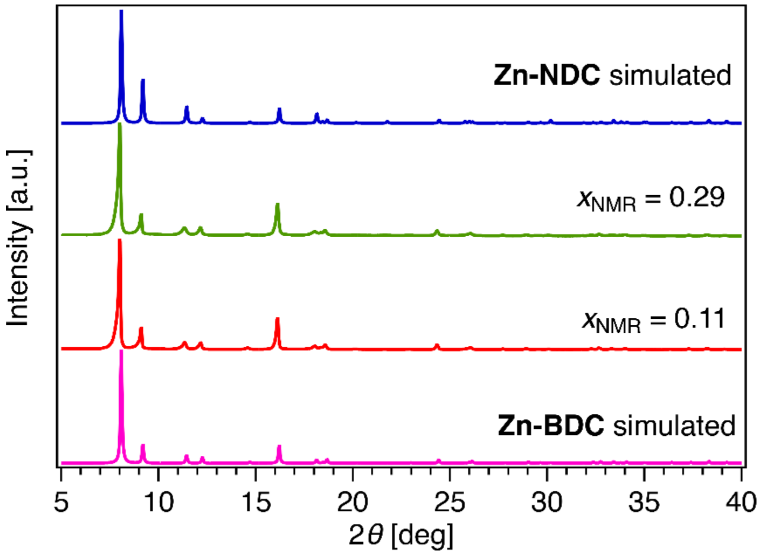

Figure S18. PXRD data of **Zn-NDC-on-Zn-BDC** with varying shell fractions: $x_{NMR} = 0.11$ (red) and $x_{NMR} = 0.29$ (green). Simulated PXRD patterns of pure **Zn-BDC** and **Zn-NDC** are shown as magenta and blue traces, respectively.

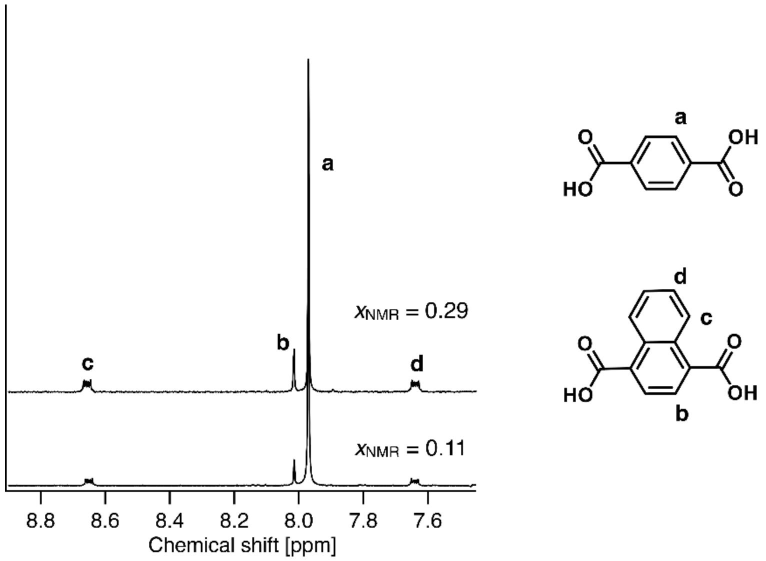

Figure S19. ^{1}H NMR spectra of aromatic protons for **Zn-NDC-on-Zn-BDC** samples with varying shell fractions: $x_{NMR} = 0.11$ (bottom) and $x_{NMR} = 0.29$ (top), measured after digestion. The samples were digested in DMSO-d_6/DCl (9/1, v/v) mixture and subsequently subjected to the NMR measurement.

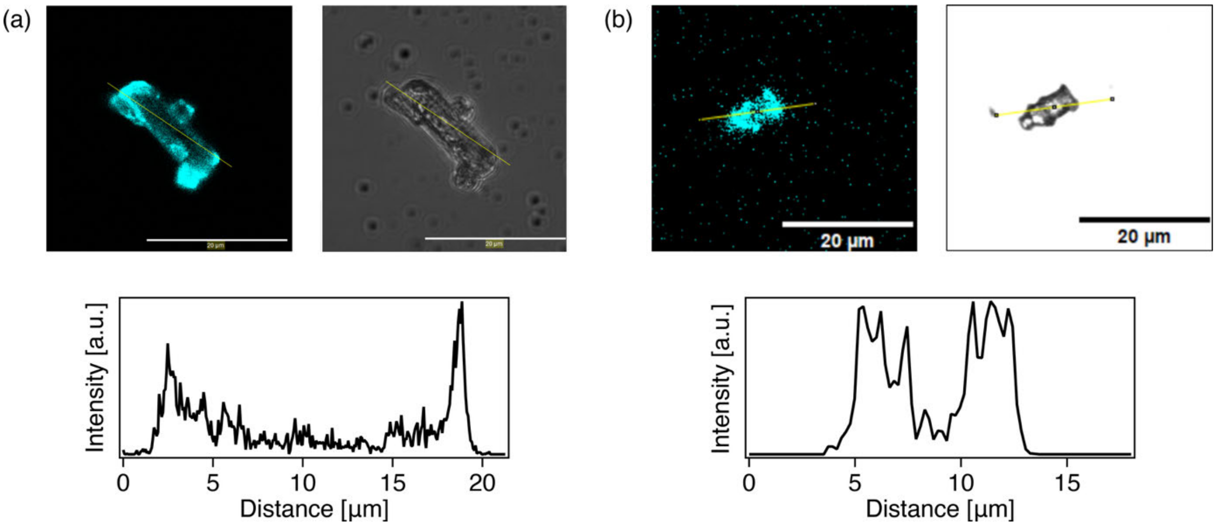

Figure S20. CLSM analysis. (a,b) Fluorescent (λ_{ex} = 405 nm) (top left) and bright field (top right) images, along with gray value line profiles (bottom) of (a) **Zn-NDC-on-Zn-BDC** (x_{NMR} = 0.11) and (b) **Zn-NDC-on-Zn-BDC** (x_{NMR} = 0.29). Scale bar: 20 µm. The gray value line profiles show the distribution of fluorescent ndc ligand along the yellow line indicated in the CLSM and bright field images (top).

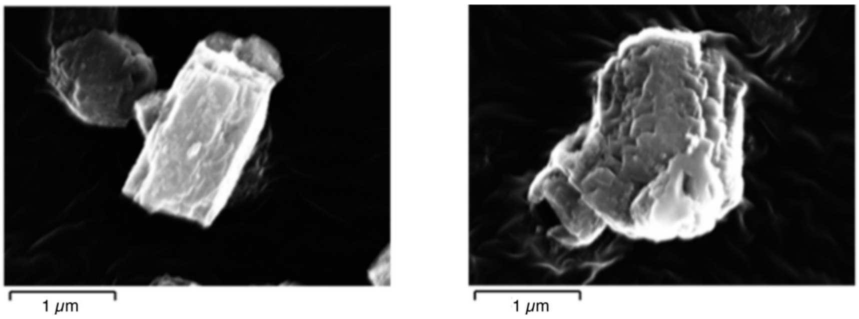

Figure S21. SEM images of representative **Cu-BDC-on-Zn-NDC** microcrystals showing deformation in shape and morphology relative to the original **Zn-NDC** core crystals after the LbL growth cycles.

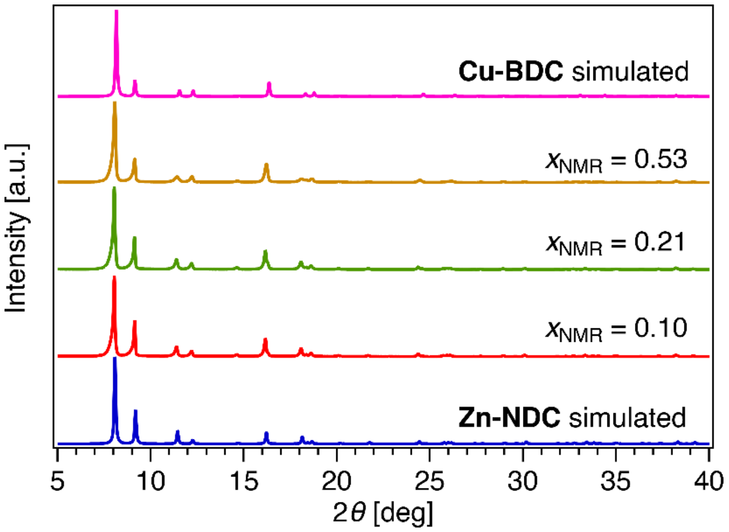

Figure S22. PXRD data of **Cu-BDC-on-Zn-NDC** with varying shell fractions: x_{NMR} = 0.10 (red), x_{NMR} = 0.21 (green), and x_{NMR} = 0.53 (khaki). Simulated PXRD patterns of pure **Cu-BDC** and **Zn-NDC** are shown as magenta and blue traces, respectively.

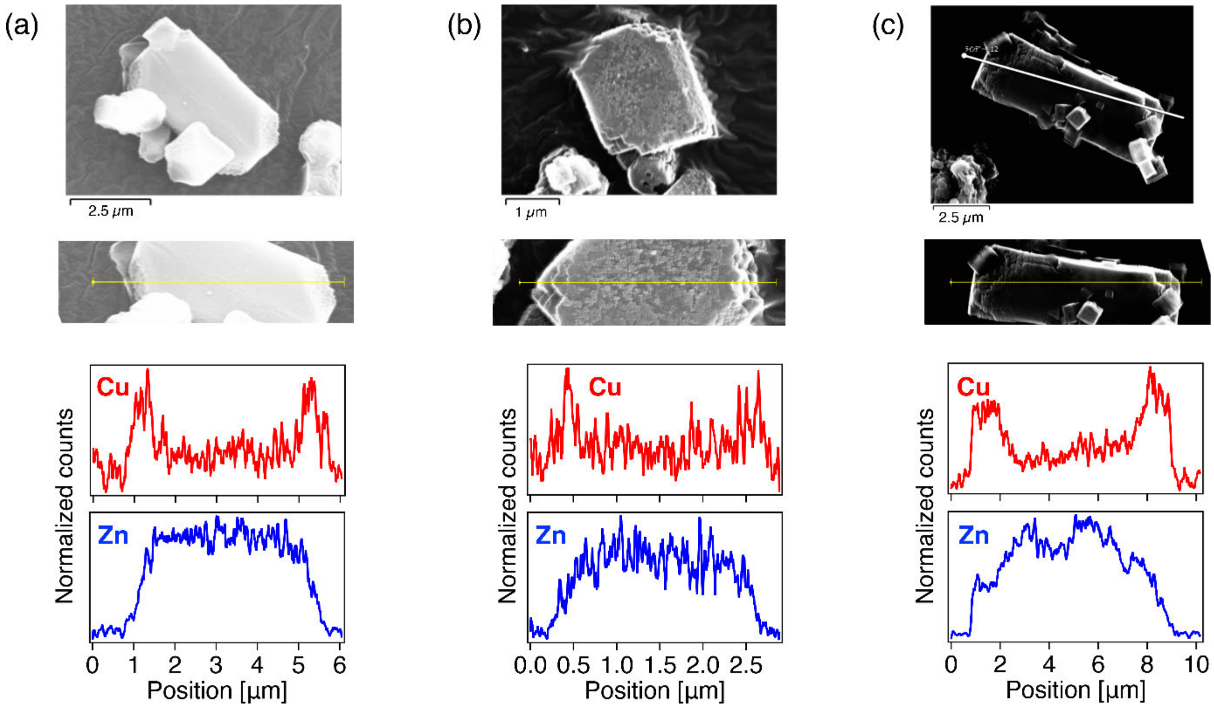

Figure S23. (a–c) SEM images (top, middle) and EDX line-scanning profiles (bottom) of **Cu-BDC-on-Zn-NDC** microcrystals with varying shell fractions: (a) x_{NMR} = 0.10, (b) x_{NMR} = 0.21, and (c) x_{NMR} = 0.53. EDX line-scan profiles show the distribution of Cu (red) and Zn (blue) along the yellow line indicated in the corresponding SEM image (middle).

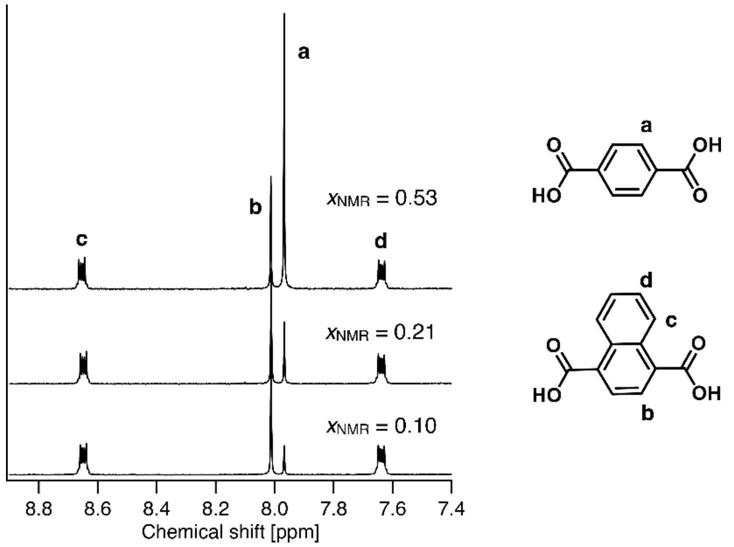

Figure S24. ^{1}H NMR spectra of aromatic protons for **Cu-BDC-on-Zn-NDC** samples with varying shell fractions: x_{NMR} = 0.10 (bottom), x_{NMR} = 0.21 (middle), and x_{NMR} = 0.53 (top), measured after digestion. The samples were digested in DMSO-d_6/DCl (9/1, v/v) mixture and subsequently subjected to the NMR measurement.

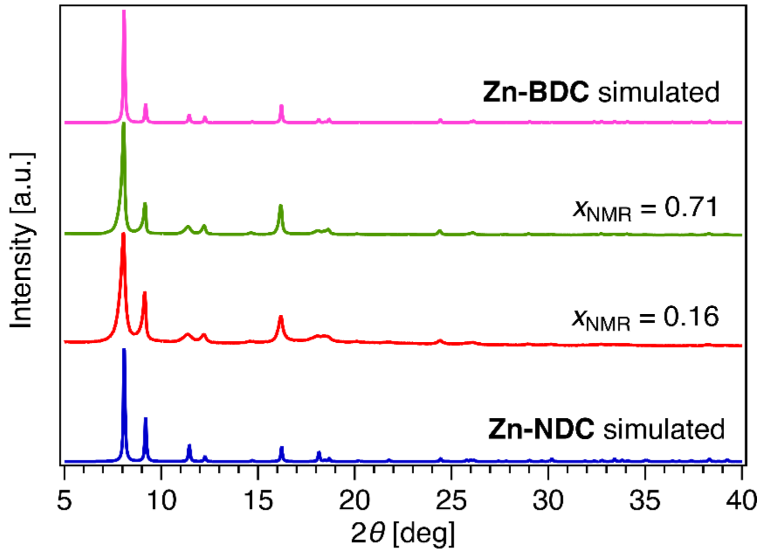

Figure S25. PXRD data of **Zn-BDC-on-Zn-NDC** with varying shell fractions: x_{NMR} = 0.16 (red) and x_{NMR} = 0.71 (green). Simulated PXRD patterns of pure **Zn-BDC** and **Zn-NDC** are shown as magenta and blue traces, respectively.

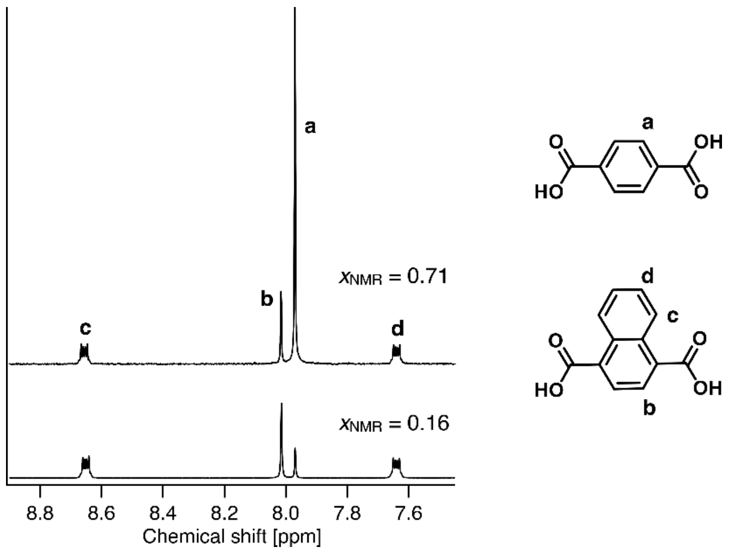

Figure S26. ^{1}H NMR spectra of aromatic protons for **Zn-BDC-on-Zn-NDC** samples with varying shell fractions: x_{NMR} = 0.16 (bottom) and x_{NMR} = 0.71 (top), measured after digestion. The samples were digested in DMSO-d_6/DCl (9/1, v/v) mixture and subsequently subjected to the NMR measurement.

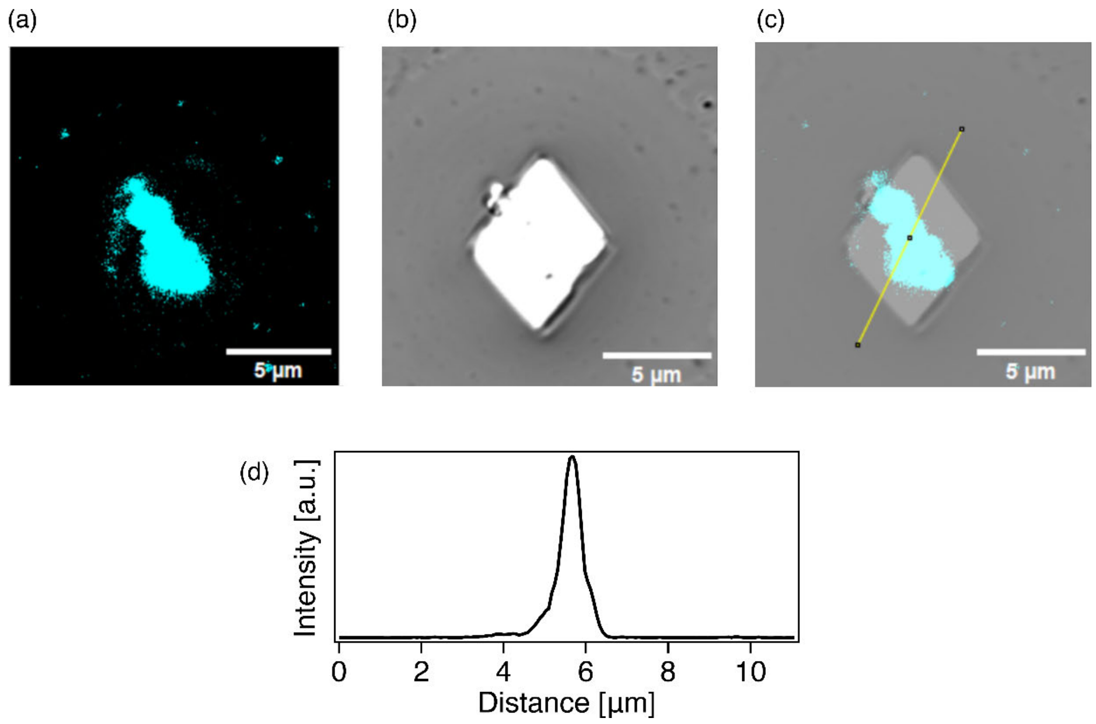

Figure S27. CLSM analysis. (a) Fluorescent (λ_{ex} = 405 nm), (b) bright field, (c) and overlay images, along with (c) gray value line profiles of **Zn-BDC-on-Zn-NDC** (x_{NMR} = 0.71). Scale bar: 5 μm. The gray value line profile shows the distribution of fluorescent ndc ligand along the yellow line indicated in panel (c). The overlay image clearly shows the **Zn-NDC** domain confined to the middle region, enclosed on both sides by the non-fluorescent **Zn-BDC** layers.

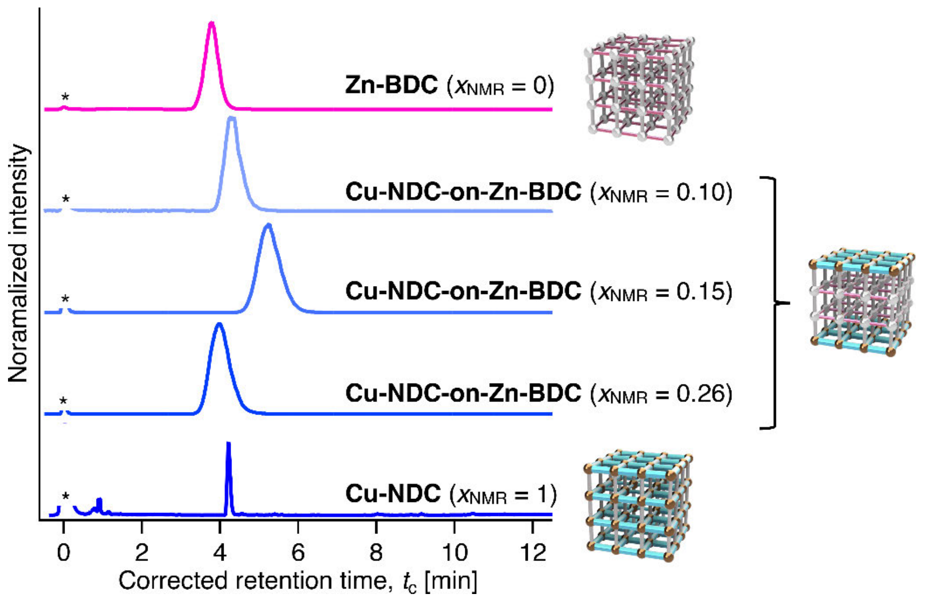

Figure S28. HPLC chromatograms of anthracene on **Cu-NDC-on-Zn-BDC** columns with varying shell fractions (x_{NMR} = 0.10, 0.15, and 0.26). Columns with x_{NMR} = 0 and 1 correspond to the parent MOFs **Zn-BDC** and **Cu-NDC**, respectively. Eluent: hexane, temperature: 40 °C, flow rate: 1.0 ml min^{-1}, detector: ELSD.

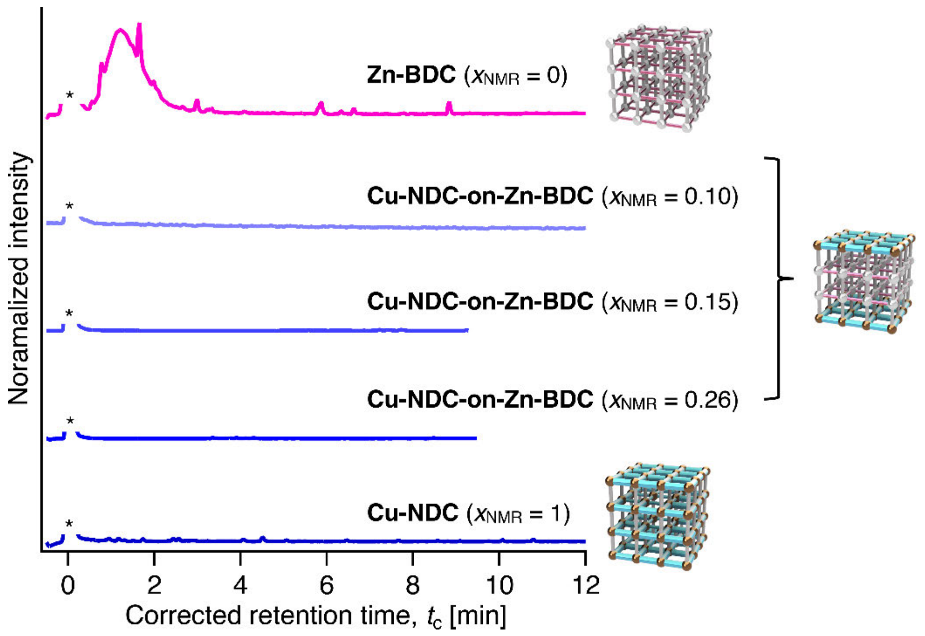

Figure S29. HPLC chromatograms of phenanthrene on **Cu-NDC-on-Zn-BDC** columns with varying shell fractions (x_{NMR} = 0.10, 0.15, and 0.26). Columns with x_{NMR} = 0 and 1 correspond to the parent MOFs **Zn-BDC** and **Cu-NDC**, respectively. Eluent: hexane, temperature: 40 °C, flow rate: 1.0 ml min^{-1}, detector: ELSD.

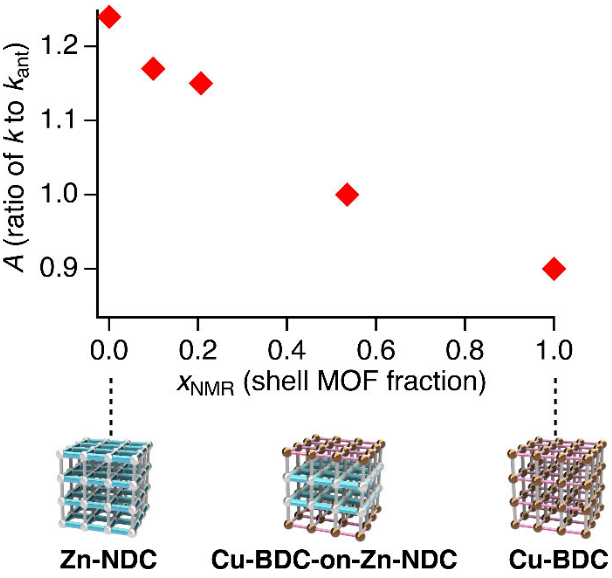

Figure S30. Retention factor ratio A for phenanthrene, measured on **Cu-BDC-on-Zn-NDC** columns, plotted as a function of shell fraction x_{NMR}. A value is defined as k/k_{ant}, where k is the retention factor of the analyte (perylene or phenanthrene) and k_{ant} is the retention factor of anthracene used as the reference material.

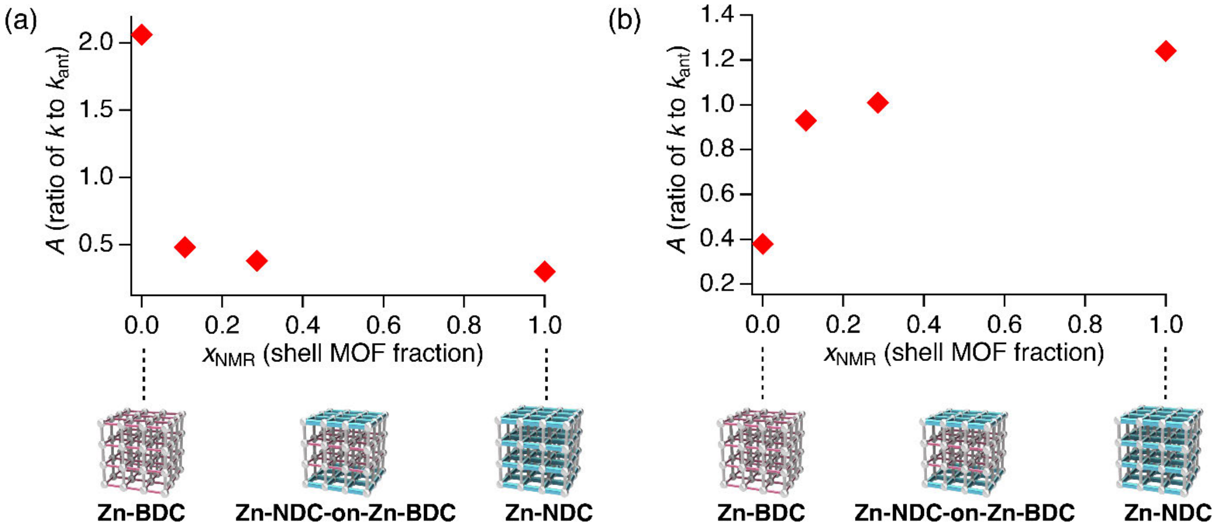

Figure S31. Retention factor ratio A for (a) perylene and (b) phenanthrene, measured on **Zn-NDC-on-Zn-BDC** columns, plotted as a function of shell fraction x_{NMR}. A value is defined as k/k_{ant}, where k is the retention factor of the analyte (perylene or phenanthrene) and k_{ant} is the retention factor of anthracene used as the reference material.

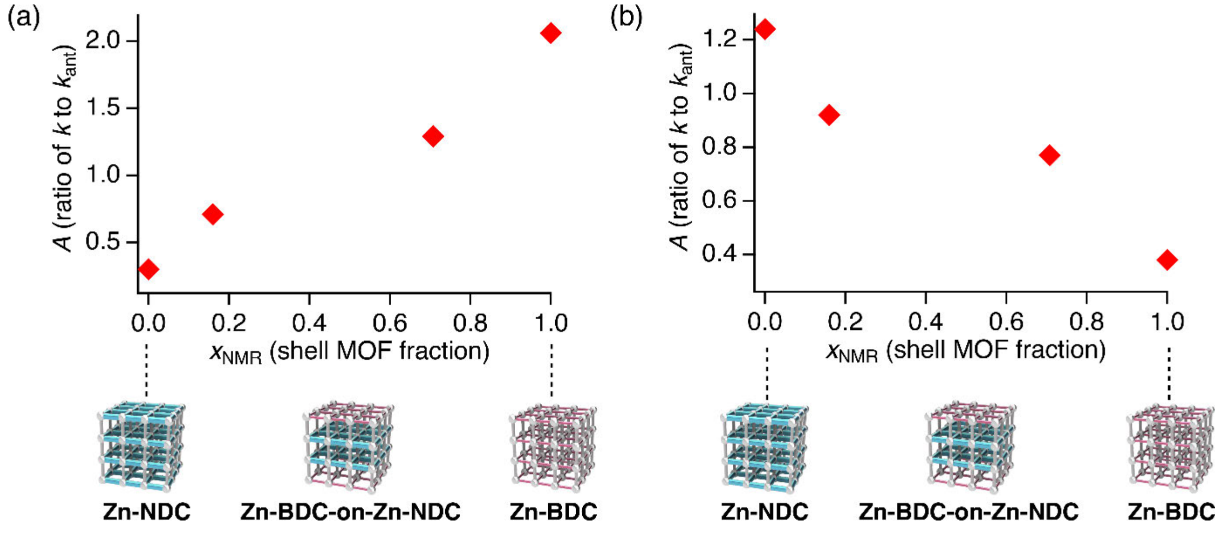

Figure S32. Retention factor ratio A for (a) perylene and (b) phenanthrene, measured on **Zn-BDC-on-Zn-NDC** columns, plotted as a function of shell fraction x_{NMR}. A value is defined as k/k_{ant}, where k is the retention factor of the analyte (perylene or phenanthrene) and k_{ant} is the retention factor of anthracene used as the reference material.

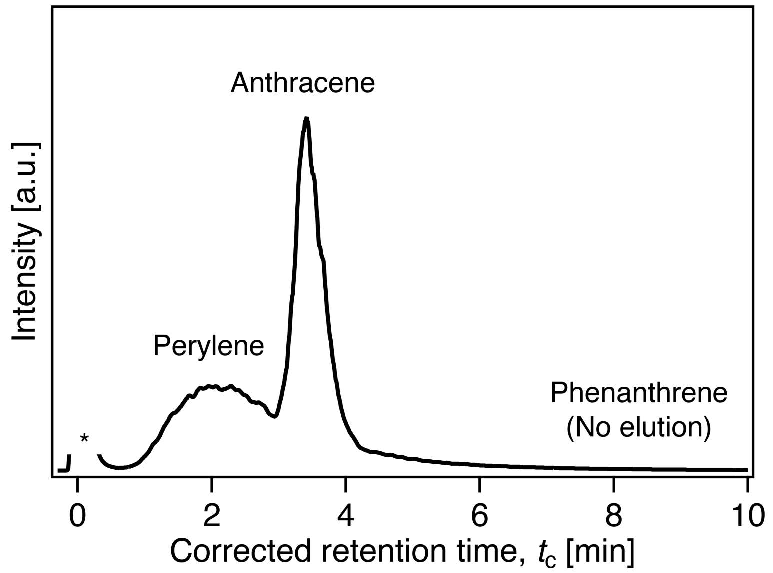

Figure S33. A HPLC chromatogram of a mixture of anthracene, perylene, and phenanthrene measured using the representative **Cu-NDC-on-Zn-BDC** (0.15) column. The chromatogram showed a separation trend consistent with the individual chromatographic runs for each analyte. Perylene and anthracene eluted at retention times similar to those observed in their respective single-component measurements, whereas phenanthrene was not eluted from the column, in agreement with its individual behavior. Eluent: hexane, temperature: 40 °C, flow rate: 1.0 ml min^{-1}, detector: ELSD.

7. Supporting References

[1] D. N. Dybtsev, H. Chun, and K. Kim. *Angew. Chem., Int. Ed.*, 2004, **43**, 5033–5036.

[2] N. Mizutani, N. Hosono, B. Le Ouay, T. Kitao, R. Matsuura, T. Kubo, and T. Uemura, *J. Am. Chem. Soc.*, 2020, **142**, 3701–3705.

[3] N. Oe, N. Hosono, and T. Uemura, *Chem. Sci.*, 2021, **12**, 12576–12586.

[4] K. Kioka, N. Mizutani, N. Hosono, and T. Uemura, *ACS Nano*, 2022, **16**, 6771–6780.

[5] H. Chun, D. N. Dybtsev, H. Kim, and K. Kim, Chem. Eur. J., 2005, **11**, 3521–3529.

[6] O. Shekhah, H. Wang, S. Kowarik, F. Schreiber, M. Paulus, M. Tolan, C. Sternemann, F. Evers, D. Zacher, R. A. Fischer, and C. Wöll, *J. Am. Chem. Soc.*, 2007, **129**, 15118–15119.

[7] K. Ikigaki, K. Okada, Y. Tokudome, T. Toyao, P. Falcaro, C. J. Doonan, and M. Takahashi, *Angew. Chem., Int. Ed.*, 2019, **58**, 6886–6890.
